# *PiggyBac* Transposon Mining in the Small Genomes of Animals

**DOI:** 10.3390/biology13010024

**Published:** 2023-12-31

**Authors:** Mengke Guo, George A. Addy, Naisu Yang, Emmanuel Asare, Han Wu, Ahmed A. Saleh, Shasha Shi, Bo Gao, Chengyi Song

**Affiliations:** 1 College of Animal Science & Technology, Yangzhou University, Yangzhou 225009, China; mx120210877@stu.yzu.edu.cn (M.G.); addysquare1@gmail.com (G.A.A.); dx120180101@yzu.edu.cn (N.Y.); asare.emmanuel175@yahoo.com (E.A.); elemlak1339@gmail.com (A.A.S.); mx120190668@yzu.edu.cn (S.S.); bgao@yzu.edu.cn (B.G.); 2 Department of Immunology, School of Medicine, Shenzhen University, Shenzhen 518060, China; wuhan@szu.edu.cn; 3 Animal and Fish Production Department, Faculty of Agriculture (Alshatby), Alexandria University, Alexandria City 11865, Egypt

**Keywords:** transposon, *piggyBac*, evolution

## Abstract

**Simple Summary:**

Transposable elements (TEs) are mobile genetic elements that play vital role in defining and contributing to the size, shape and structure of both prokaryotic and eukaryotic genomes in nature. The *piggyBac* (*PB*), a superfamily of DNA transposons, has been isolated over the years from various organisms including insects, fungi and plants. These *piggyBac* transposon systems have high efficiency with a wide usage in the study of gene therapies, mutagenesis and transgenesis. Currently, there is limited information available on DNA transposons in small (compact) genomes of animals. Therefore, this study aims to annotate the *PB* transposons in small genomes of animals, revealing their evolution profiles in both vertebrate and invertebrate genomes.

**Abstract:**

TEs, including DNA transposons, are major contributors of genome expansions, and have played a very significant role in shaping the evolution of animal genomes, due to their capacity to jump from one genomic position to the other. In this study, we investigated the evolution landscapes of *PB* transposons, including their distribution, diversity, activity and structure organization in 79 species of small (compact) genomes of animals comprising both vertebrate and invertebrates. Overall, 212 *PB* transposon types were detected from almost half (37) of the total number of the small genome species (79) investigated. The detected *PB* transposon types, which were unevenly distributed in various genera and phyla, have been classified into seven distinct clades or families with good bootstrap support (>80%). The *PB* transposon types that were identified have a length ranging from 1.23 kb to 9.51 kb. They encode transposases of approximately ≥500 amino acids in length, and possess terminal inverted repeats (TIRs) ranging from 4 bp to 24 bp. Though some of the transposon types have long TIRs (528 bp), they still maintain the consistent and reliable 4 bp target site duplication (TSD) of TTAA. However, PiggyBac-2_Cvir transposon originating from the *Crassostrea virginica* species exhibits a unique TSD of TATG. The TIRs of the transposons in all the seven families display high divergence, with a highly conserved 5′ end motif. The core transposase domains (DDD) were better conserved among the seven different families compared to the other protein domains, which were less prevalent in the vertebrate genome. The divergent evolution dynamics analysis also indicated that the majority of the *PB* transposon types identified in this study are either relatively young or old, with some being active. Additionally, numerous invasions of *PB* transposons were found in the genomes of both vertebrate and invertebrate animals. The data reveals that the *PB* superfamily is widely distributed in these species. *PB* transposons exhibit high diversity and activity in the small genomes of animals, and might play a crucial role in shaping the evolution of these small genomes of animals.

## 1. Introduction

Transposable elements (TEs) are mobile units in genomes; they play vital role in defining and contributing to the size, shape and structure of both prokaryotic and eukaryotic genomes in nature [1]. TEs are classified into two categories, based on their transposable mechanisms. Class I, retrotransposons, rely on RNA intermediates and undergo transposition through a copy and paste mechanism. On the other hand, Class II, DNA transposons, employ DNA intermediates in a mechanism known as a cut and paste system [2]. Several super families of DNA transposons, including *PB*, *Tc1/mariner*, *pogo*, *hAT*, *Helitron* and *PIF-Harbinger*, have been reported. However, only the *Tc1/mariner* and *pogo* DNA transposon families have a well-documented evolutionary history. Although TEs do not rely on a sequence relationship between donors and recipients, they have been identified in various organisms and comprise approximately 35–69% of vertebrate genomes [3]. TEs constitute a significant portion of the nuclear DNA content, and generate genetic diversity at the sequence, gene structure and chromosomal levels. Animals, as a highly successful taxon, exhibit lineage-specific variations in TE content, implying diverse impacts on the compact genomes [4]. TEs’ existence are utilized as genetic elements for analyzing gene expression, protein functions, and genetic engineering. A recent catalog of transposon sequences in eukaryotic organisms identified well-characterized transposon genes in animal genomes, with several families sharing similar sequences across closely and distantly related compact genomes [5].

The *PB* transposon is a transferable genetic component which efficiently transposes through a “cut-and-paste” mechanism [6]. The *PB* transposons are used for efficient gene transfer tools in animals such as mice, rats, and rabbits. Aquatic creatures such as sea anemones, eels, marine worms, sea cucumbers, small crustaceans, snails, sculpin and urchins have been acknowledged to contain *PB* transposons [7]. Most members of the *PB* super family use the four-base pair (TTAA) tetranucleotides as their target sites of duplications [8]. The *PB* transposons also transpose in organisms such as yeasts, malaria parasites, insects, etc. with a molecular mass of 64 kDa [9]. A well-defined *PB* transposon is about 2472 bp in length, with two terminal inverted repeat sequences and a transposase encoding domain sequence [10].

The smooth pufferfishes, *Lagocephalus laegavigatus*, belong to the order Tetraodontiformes, and comprises of highly derived ray-finned fish. They represent the smallest fraction of the compact genome of animals measured to date [11,12,13,14]. They descended from a line of coral-dwelling species that emerged around 80 million years ago [15]. *Tetraodontiforms* make up about five percent of the tropical marine vertebrates with a wide range of both morphological and ecological diverse radiations [16]. The smooth pufferfish are widely known to be poisonous and lethal to consume, due to the presence of the neurotoxins saxitoxin and tetrodotoxin substances found in their gonads [17]. According to Shao et al. (2019), the differences in genome size to the variation in TE content (5–56%) across fish species and other vertebrates plays important role in evolution of compact genomes [18]. Comparative genomic analysis revealed that the DNA transposons are the major contributors of genome size differences between the four teleost genomes (zebrafish, medaka, stickleback and tetraodon) [19]. However, very little information has been reported about the evolution profile of DNA transposons in the compact genomes of animals. In this study, we examined *PB* transposons in the compact genomes of various animals. We annotated the *PB* transposons in each species’ genome to identify their structural characteristics, distribution patterns and classifications, and conducted an analysis of their evolutionary dynamics. Our findings provide insight into the evolutionary patterns of *PB* transposons in the small genomes of animals, to enhance the understanding of their contribution to the animal genome.

## 2. Materials and Methods

### 2.1. PB Transposon Mining

Ten vertebrate genomes of Tetraodontiformes, eight compact genomes of *Tetradontoidea* species (*Arothron firmamentum*, *Lagocephalus sceleratus*, *Pao palembangensis*, *Takifugu bimaculatus*, *Takifugu flavidus*, *Takifugu ocellatus*, *Takifugu rubripes*, and *Tetraodon nigroviridis*), as well as the relatively large genomes of *Mola mola* and *Thamnaconus septentrionalis*, which belong to the sister group of *Tetraodontidae* [20], were retrieved from the NCBI database (Table S1). For invertebrates, the compact genomes of invertebrates were chosen based on their C-values obtained from the database of genome size (http://www.genomesize.com/, accessed on 13 June 2023). First, the C-values for each species within various lineages (Annelids, Arachnids, Crustaceans, Echinoderms, Flatworms, Insects, Mollusks, and Nematodes) were extracted from the genome size database (http://www.genomesize.com/, accessed on 13 June 2023) and ranked. Subsequently, the ten species with the smallest C-values in each lineage (a total of 80 species) were selected. We retrieved the available assembled genomes of these species from the NCBI genome database. Finally, we obtained a total of 69 genomes with the smallest C-values, referred to as the “compact genomes” of each lineage, representing ten lineages: seven for Annelids, ten for Arachnids, ten for Crustaceans, six for Echinoderms, six for Flatworms, ten for Insects, ten for Mollusks, and ten for Nematodes. These lineages belong to six invertebrate phyla, including seven Annelida species, 30 Arthropoda species (ten Arachnids, ten Crustaceans, and ten Insects), ten Nematoda species, six Platyhelminthes species, ten Mollusca species, and six Echinodermata species. We selected the best assembled genome for each species (listed in Table S1) and performed local TBLASTN analysis.

*PB* transposons were identified by performing a local TBLASTN search on downloaded genomes. The queries used were the DDE domains of *PB* transposases, with a cutoff value of 1 × 10^−4^. Genomic sequences with over 30% coverage and over 80% identity from TBLASTN hits were extracted, along with 4 kb upstream and downstream flanking sequences. The obtained sequences from all genomes were then clustered using the USEARCH program, with a 50% identity threshold. Subsequently, the MAFFT program was used to align the sequences and identify the transposon boundaries, including terminal inverted repeats (TIRs) and target site of duplications (TSDs) [21]. The presence of TSDs and TIRs in all sequences and alignments was manually confirmed. To determine the copy number of each type of transposon in each species, BlastN was employed with criteria of over 40% coverage and over 80% identity. Elements displaying detectable TIRs and TSDs of *PB* sequences were selected and classified as *PB* transposons, while sequences flanked by only one TIR or one TSD were considered *PB* transposon-like sequences.

### 2.2. Phylogenetic Tree Construction

The putative full-length transposase sequences (larger than 500 amino acids) obtained from each transposon, along with reference sequences of *Pokey* and representative prokaryote IS1380 transposases from ISfinder (https://www-is.biotoul.fr/index.php, accessed on 1 September 2023), were aligned using the G-INS-I method in MAFFT software (v. 7.310) [21]. The resulting alignments were used to construct a phylogenetic tree using the IQ-tree program [22]. The ultrafast bootstrap approach with 1000 replicates was applied. The appropriate amino acid substitution model was determined using ModelFinder [23]. The IS1380 transposases were used as the outgroup.

### 2.3. Evolutionary Dynamics Analysis

The evolutionary dynamics of *PB* transposons in each genome were assessed using the Kimura (K) divergence based on the RepeatMasker program. The calculation of K divergence was performed using the “calcDivergenceFromAlign.pl” package from RepeatMasker [24]. This measure offers valuable insight into the relative activity of transposons within each genome over a specific time frame [25].

### 2.4. PB Sequence Analysis

The potential open reading frames (ORFs) in the obtained sequences were predicted using GENSCAN (http://hollywood.mit.edu/GENSCAN.html, accessed on 10 August 2023). The protein domains were identified using profile hidden Markov models through the hmmscan web server (https://www.ebi.ac.uk/Tools/hmmer/search/hmmscan, accessed on 20 August 2023). The structure of the *PB* transposase was illustrated using the Illustrator for Biological Sequences (v. 1.0.3) [26]. Multiple alignments were performed using MAFFT (v. 7.310) and visualized using Jalview Version 2. The TIR, DDD and DDBD1 sequences of *PB* elements or transposases were aligned using MAFFT (v. 7.310), and the sequence identities were calculated using the BioEdit tool (v. 7.2.0) [27]. The sequence identities obtained were visualized using the HeatMap program in GraphPad Prism (v. 8.0.2). Additionally, sequence logos were created using TBtools (v1.0987663) (https://github.com/CJ-Chen/TBtools/releases, accessed on 12 September 2023) [28].

## 3. Results

### 3.1. Distribution of PB Transposons in the Compact Genomes of Animals

Genomes with relatively small or compact sizes were defined based on the ranking C-values obtained from the genome size database (http://www.genomesize.com/, accessed on 13 June 2023) for each lineage, which include Annelids, Arachnids, Crustaceans, Echinoderms, Flatworms, Insects, Mollusks, Nematodes and Tetradontoidea, as described in the Materials and Methods section. These genomes were subsequently downloaded from the NCBI database for further analysis of *PB* annotation. The examined compact genomes include eight vertebrate species and 69 invertebrate species, representing seven different phyla: Annelida, Arthropoda, Chordata, Echinodermata, Mollusca, Platyhelminthes and Nematoda. As controls, the relatively large genomes of *Mola mola* and *Thamnaconus septentrionalis* were included from the vertebrate species. Echinoderms and Platyhelminthes contributed six species each, the lowest fraction among the studied groups. Annelida contributed seven species, Arthropoda contributed the highest fraction with 30 species, while Chordata, Mollusca and Nematoda contributed ten species each.

Based on the mining protocol described in methods, overall, *PB* displays high diversity and wide distribution in compact genomes, out of the 37 *PB* transposon containing species, we deduced that six *PB* transposon species each were obtained from the phyla Chordata and Annelida forming 32.4%. Echinodermata and Platyhelminthes contain two *PB* transposons species, each making up 10.8%. Mollusca contain eight *PB* transposons species, representing 21.6%, and 13 *PB* transposon species were also obtained from Arthropoda, accounting for 35.1%. Nevertheless, no *PB* transposon species has been found in the phylum of Nematoda, representing zero percent, as shown in Table 1 below.

A total of 212 different *PB* transposon types were detected from 37 species. They were irregularly distributed across different phyla/lineages of both vertebrate and invertebrate species. Seven species only contain one *PB* transposon type in their genomes, while 30 species contain more than one *PB* transposon types, which were recognized by different TIRs, or TSDs, or transposases (Table 1 and Table S2) Out of the 79 species examined, 15 *PB* transposon types were detected from the vertebrate genome of Tetraodontiformes, and *Takifugu bimaculatus* contain four *PB* transposon types, representing the highest fraction among the vertebrates. In contrast, very few *PB* transposons invaded relatively large vertebral genomes of *Thamnaconus septentrionalis* and *Mola mola*, as shown in Table S2.

Further investigation revealed a significant diversity and widespread distribution of *PB* transposons within the compact genomes of invertebrates. The remaining 197 *PB* transposon types detected were isolated from the genomes of invertebrates, with *Apporectodea caliginosa* having the highest *PB* transposon types (28). *Daphnia pulex*, *Eisenia fetida*, *Hirudo medicinalis*, and *Pinctada fucata* have the least (one) *PB* transposons each. *Acanthopleura granulata*, *Archegozetes longisetosus*, *Daphnia pulicaria*, *Lepeophtheirus salmonis* and *Lytechinus variegatus* contributed two *PB* transposon types each. Significant number of *PB* transposon types were also acknowledged from *Argiope trifasciata*, *Girardia tigrina*, *Argiope aurantia*, *schmidtea mediterranea* and *Lumbricus rubellus*, as they contributed 21, 21, 16, 13 and 11 *PB* transposon types, respectively.

Though a total of 212 *PB* transposon types were mined in all, only 85 *PB* transposon types contain intact copy of TIRs, TSDs and encode a transposase ≥ 500 aa, suggesting that these *PB* transposon types might have invaded into the genomes very recently so they maintain their transposition activities. Three *PB* transposon types harboring four intact *PB* copies were obtained from the vertebrate genome. A total of 81 *PB* transposon types were also obtained from the invertebrate genome, with 26 intact *PB* copies each from Annelida, and Arthropoda, and 19 intact *PB* copies from Mollusca, while Platyhelminthes, Chordata and Echinodermata contributed seven, four and three intact *PB* copies, respectively. However, 127 *PB* transposon types discovered did not contain any intact copies of *PB* transposon.

In addition, we analyzed the number of *PB* transposons types detected (diversity), the number of copies (abundance), and the number of intact *PB* copies (activity) in each genome. Supplementary Figure S1 provides a visualization of these relationships. The analysis revealed that there might be a positive correlation between the diversity of *PB* transposons and genome size, suggesting that larger genomes might have a higher diversity of *PB* transposons. However, we did not observe any correlation between the abundance or activity of *PB* transposons and genome size. Overall, animals with small genomes exhibit a wide distribution, high diversity, and activity of *PB* transposons.

### 3.2. Structural Organization and Classification of PB in the Compact Genomes of Animals

Substantial variations in the full-length of the *PB* transposons were observed, ranging from 1.23 kb to 9.51 kb. The transposons detected contain single ORFs and encode transposases that are flanked by both TIRs and TSDs, as shown in Table 1. Though the *PB* transposons discovered encrypt transposases between 54 aa to 1316 aa, the total transposases encoded by the intact *PB* transposons ranges between 503 aa to 1316 aa. Most of intact transposases are between 503 aa to 675 aa in lengths, whilst seven *PB* transposons types of the examined species are truncated, and therefore do not encode for any transposase. Species such as *Aporrectodea caliginosa*, *Argiope trifasciata*, *Girardia tigrina*, *Loxosceles reclusa*, *Pao palembangensis*, *Takifugu ocellatus* and *Takifugu bimaculatus* have very short *PB* transposon lengths, which range from 1.23 kb to 1.51 kb, with the shortest being *Takifugu bimaculatus* and *Girardia tigrina*, having 1234 bp and 1291 bp, respectively. However, other species such as *Argiope aurantia*, *Argiope trifasciata*, *Daphnia obtuse*, *Daphnia pulicaria*, *Crassostrea virginica*, *Crassostrea gigas* and *Lytechinus variegatus* contain *PB* transposons with longer lengths, ranging from 5.60 kb to 9.51 kb, with *Argiope aurantia* harboring the longest *PB* transposon length. The *Argiope aurantia*, which contains the longest *PB* transposon length, has a CDS sequence of 1635 bp and encodes a transposase length of >500 aa with an intact copy number of only two. Most of the *PB* transposons attained from the analysis carried short TIRs (<20 bp). Species like *Callinectes sapidus*, *Lytechinus variegatus*, *Biomphalaria glabrata*, *Argiope trifasciata*, *Tetragnatha versicolor* and *Latrodectus hesperus* have very short TIRs, with the lengths ranging from 4 to 6 bp. Nevertheless, a few species, such as *Aporrectodea caliginosa*, *Callinectes sapidus*, *Crassostrea virginica*, *Girardia tigrina*, *Lumbricus rubellus*, *mytilisepta virgata* and *Takifugu bimaculatus*, harbor long TIRs, ≥23 bp, with *Crassostrea virginica* carrying the longest TIR (528 bp). Only eight *PB* transposon elements do not maintained the conserved tetra nucleotides base pair of TTAA, with seven other *PB* transposons having no ORFs because they are condensed and truncated.

**Figure 1:**
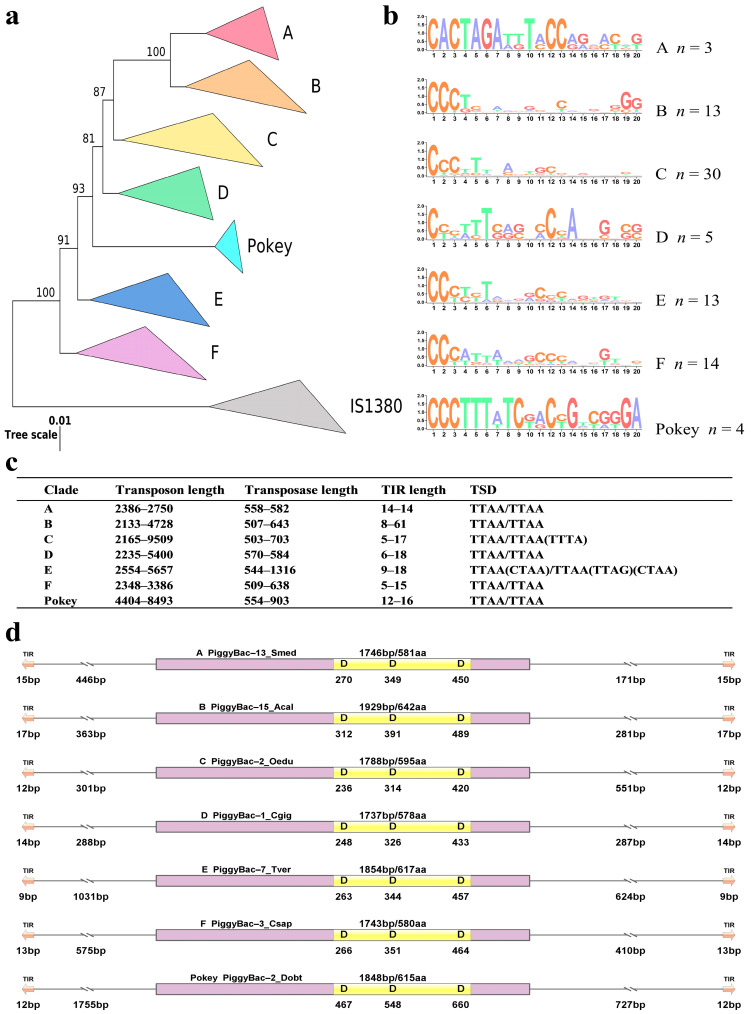
Classification and structural organization of *piggyBac* (*PB*). (**a**) Phylogenetic tree of *PB* elements in animals identified in our study, together with 82 reference sequences. The sequences of IS1380 were used as the outgroup. Red, Orange, Yellow, Green, Cyan, Blue, Purple and Grey, respectively, stand for seven types of *PB* transposons (A–F and *Pokey*). The complete evolutionary tree is as shown in Figure S2. (**b**) The alignment logo for *PB* elements. The TBtools (v1.0987663) (https://github.com/CJ-Chen/TBtools/releases, accessed on 12 September 2023) was used to create the logo representation of the first 20 bp of the TIR 5′ sequences. (**c**) Information for each clade. (**d**) Structure organization summary table of families. The orange arrows represent TIR, yellow column represents DDD domains, and purple column represents transposons.

The IQ-tree program was used to construct the evolutionary tree for the extracted *PB* transposases (≥500 aa) found with 82 reference sequences from eukaryotes, and the IS1380 transposases from prokaryotes were set as an outgroup [22]. The deduced phylogenetic tree revealed seven distinct clades (A, B, C, D, E, F and *Pokey*) of *PB* transposons in the genomes of animals with robust bootstrap supports (>80%), as shown in Figure 1a. From the analysis, it was revealed that most *PB* naked elements have very short TIRs (<20 bp) and very few *PB* transposons, such as PiggyBac-17_Acal and PiggyBac-2_Cvir from *Aporrectodea caliginosa* and *Crassostrea virginica* species, respectively, have TIRs that are up to 61 bp and above in length, up to 528 bp (Table 1 and Table S2). Remarkably, an assessment of the generalized TIR sequence-logo of the *PB* elements excavated revealed that the TIRs are equitably divergent across the seven clades. The 5′ ends of almost all the TIRs are extremely conventional with perceptibly higher cysteine content. Four different well-maintained motifs acknowledged and observed in the 5′ ends of the TIRs were CACTA, CCCTC, CCCTT and CCCAT, with the CCCTT being the most conserved motifs (Figure 1b). The consistent TSD of TTAA was observed in most of the *PB* transposon elements disclosed. However, very few of these *PB* transposons harbor different TSDs, such as TTAG, TAAA, CTAA, ATTA, TCAA, GTAA and TATG, particularly in the E clade (Figure 1c and Table S2). In addition, a comparison between the transposons and transposases across the seven clades of *PB* elements reveals that long transposases length were only observed in the clade of E (544–1316 aa) and *Pokey* (554–903 aa), while transposases from other clades range from 503 aa to 703 aa in length. Long *PB* elements were also located in clade C and *Pokey*, as shown in Figure 1c,d and Table S2.

### 3.3. Sequence Analysis across the Seven Clades of PB

**Figure 2:**
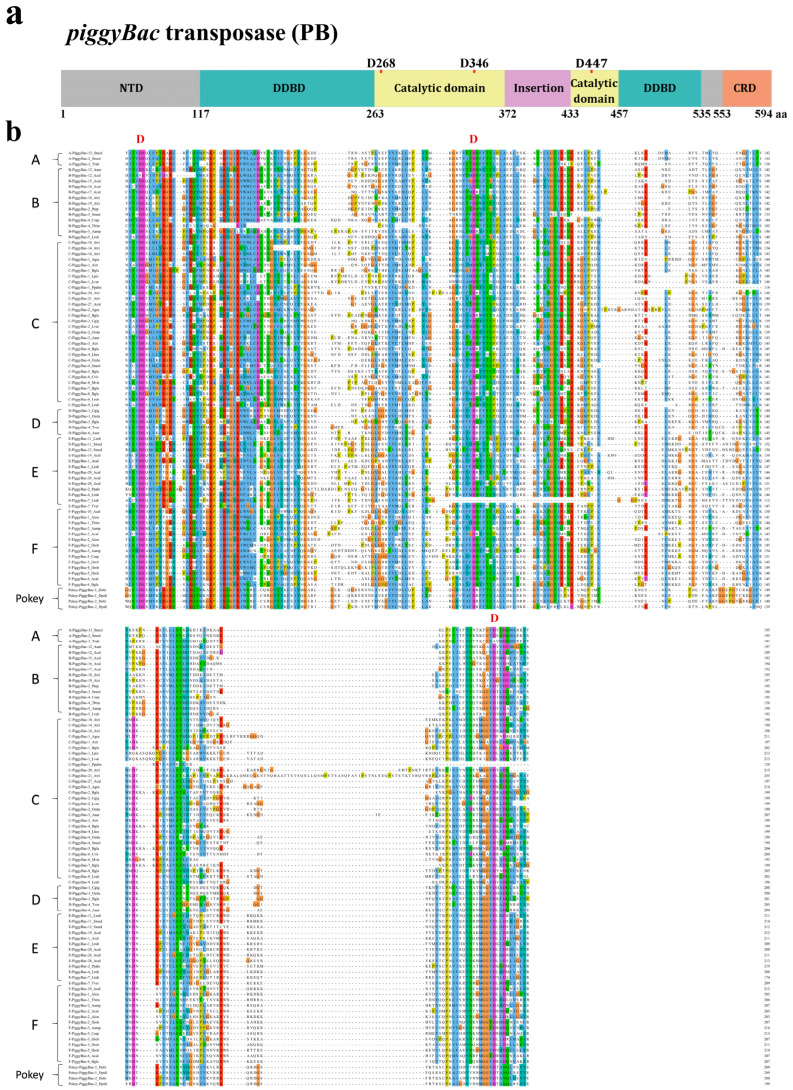
Sequence analysis. (**a**) *PB* transposases. (**b**) Multiple sequence alignments were performed on the DDD domains of complete *PB* transposases classified into seven. Multiple sequence alignments of other domains are in Supplementary Figure S3.

**Figure 3:**
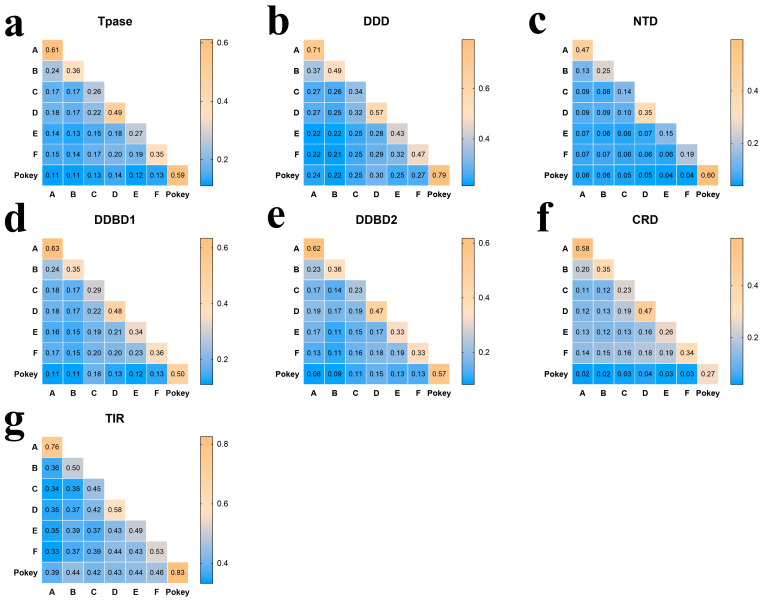
Sequence identities between A–*Pokey* clades. The numbers in the heatmap represent the percentage of the average sequence identities between the two clades of transposons. The average sequence identities were calculated by pairwise comparing of the sequences of Tpase (transposase) (**a**), DDD (Catalytic domain) (**b**), NTD (N-terminal domain) (**c**), DDBD1 (Dimerization and DNA-binding domain one) (**d**), DDBD2 (Dimerization and DNA-binding domain two) (**e**), CRD(C-terminal cysteine-rich domain ) (**f**) and TIR (Terminal inverted repeat) (**g**) from complete *PB* transposases with a length of at least 500 amino acids.

The discovered *PB* elements exposed *PB* transposases branded to encompass five major domains, which includes N-terminal domain (NTD), Dimerization and DNA-binding domain one (DDBD1), Catalytic domain (either DDD/DDE), Dimerization and DNA-binding domain two (DDBD2) and the C-terminal cysteine-rich domain (CRD) [8], as shown in Figure 2a. Differential evolution patterns were also observed for the different domains of *PB* transposases. The various catalytic domains (DDD) of the seven clades are highly conserved. Clade A and B have their first and second aspartate residues separated by 79 amino acids, clades C and D have their first and second aspartate residues acids separated by 78 amino acids, whereas 81 amino acids separated the first and second aspartate residues of clade E and *Pokey*, with clade F containing 85 amino acids to differentiate between its first and second aspartate residues. The distance between the second and third aspartate residues of the seven clades varies from 98 to 113 amino acids, as shown in Figure 1d, and the CDS length of all the seven clades ranges between 1737 and 1929 bp. The *PB* transposases exhibit a comparable level of diversity among the different clades, with sequence identities of 11–22% within clades and 26–61% between clades (Figure 3a). On the other hand, the DDD domains demonstrate a high level of sequence identity within and between the clades, ranging from 21 to 37% and 34 to 79%, respectively (Figure 3b). The three essential catalytic residues (DDD), necessary for catalyzing the transposition reaction of the transposons, are likewise highly conserved among the transposases of all seven clades examined [29]. The insertion motif located between the second and third catalytic residues is consistently conserved across all seven clades, particularly in the C-terminal insertion motifs of clade C transposases (Figure S3). The CRD, DDBD1, DDBD2 and NTD domains of the *PB* elements exhibit limited conservation, showing low sequence identities within and between the clades. Similarly, the N-terminal domain (NTD) shows remarkably low sequence identities across different clades, all of which are below 0.1 (Figure 3c–f). Moreover, the CRD that normally acts as the propelling force of the TIR binding force exhibits eight cysteine residues with regular spacing between them within the transposases from seven clades [8]. The observed residues of DDBD1 among the transposases of the seven clades are also preserved [30]. The N-terminal of DDBD2 as usual harbors tryptophan rudiments to perform an essential role in the activity of the transposase [31]. Many of the observed *PB* elements exhibited a high degree of similarity between their left and right terminal inverted repeats (TIRs), indicating a significant identity in the TIR sequence (Table S2). However, low level conservation of TIR within the same clade (33–46%) or between clades (45–83%) as shown in Figure 3g.

### 3.4. Evolution Dynamics of PB in the Compact Genomes of Animals

**Figure 4:**
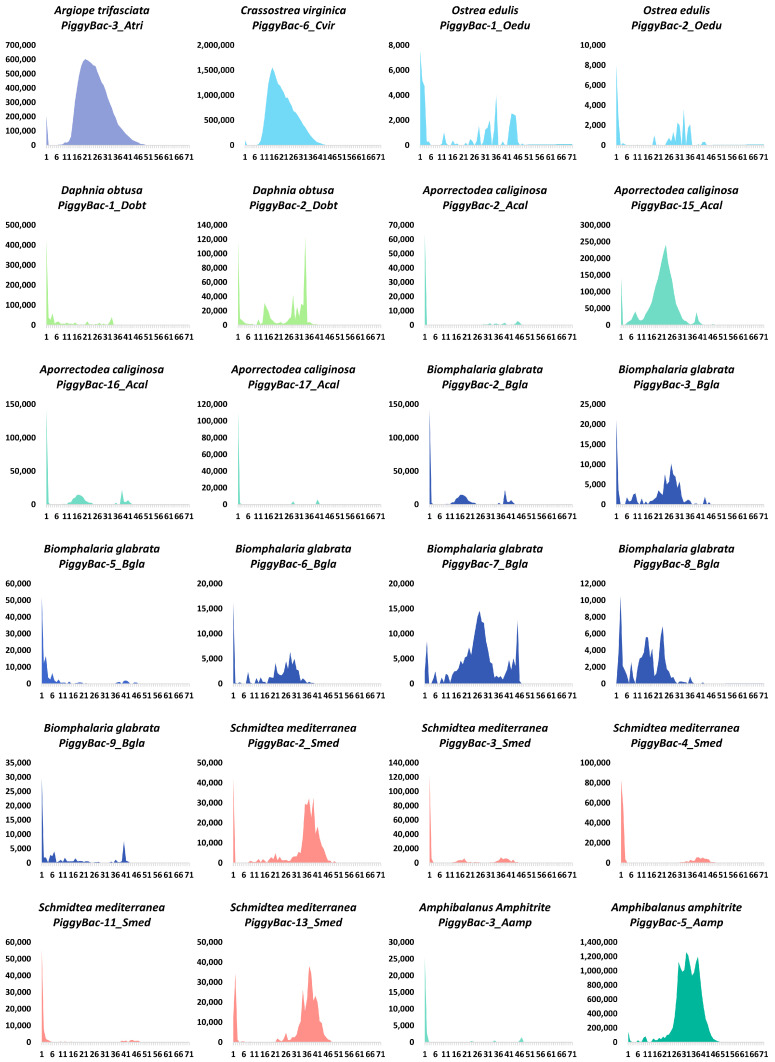
K divergences of evolutionary dynamics in compact genomes of animals. The *y*-axis represents the coverage (%) of each *PB* transposon in the genome, while the *x*-axis indicates the K divergence estimate (%). The names of the species are displayed at the top of each chart.

The Kimura (K) divergences, which reflect the insertion ages of transposons in the genome [25], were utilized to assess the evasion histories of *PB* transposons in genomes where a high number of intact copies (>6) of *PB* were detected. Differential evolutionary histories were observed for the 24 *PB* transposons in genomes (Figure 4). *PB* transposons in some genomes seems represent long and lasting invasions, such as PiggyBac-3 in *Argiope trifasciata* (Atri); PiggyBac-6 in *Crassostrea virginica* (Cvir); PiggyBac-15 in *Aporrectodea caliginosa* (Acal); PiggyBac-3, PiggyBac-6, PiggyBac-7 and PiggyBac-8 in *Biomphalaria glabrata* (Bgla); PiggyBac-2 and PiggyBac-13 in *Schmidtea mediterranea* (Smed); PiggyBac-5 in *Amphibalanus amphitrite* (Aamp); and multiple waves of amplification were observed for some *PB* transposons, such as PiggyBac-2 in *Daphina obtusa* (Dobt); PiggyBac-1 and PiggyBac-2 in *Ostrea edulis* (Oedu); PiggyBac-7 in *Biomphalaria glabrata* (Bgla); PiggyBac-8 in *Biomphalaria glabrata* (Bgla); PiggyBac-2 in *Schmidtea mediterranea* (Smed); and PiggyBac-13 in *Schmidtea mediterranea* (Smed). While the K divergences also suggest that *PB* transposons might be currently active in some species, such as PiggyBac-2 in *Ostrea edulis* (Oedu); PiggyBac-1 in *Daphina obtusa* (Dobt); PiggyBac-2, PiggyBac-16 and PiggyBac-17 in *Aporrectodea caliginosa* (Acal); PiggyBac-2 and PiggyBac-9 in *Biomphalaria glabrata* (Bgla); PiggyBac-3, PiggyBac-4 and PiggyBac-11 in *Schmidtea mediterranea* (Smed); and PiggyBac-3 in *Amphibalanus amphitrite* (Aamp), where most *PB* copies display very low levels of K divergence (close to zero) in these genomes and represent very recent genomic invasions, indicating that they may have the capability of transposition. In addition, young and old *PB* transposons co-exist in some genomes, such as *Argiope trifasciata* (Atri), *Aporrectodea caliginosa* (Acal), *Schmidtea mediterranea* (Smed), *Amphibalanus amphitrite* (Aamp), and *Biomphalaria glabrata* (Bgla), indicating that these species experienced recurrent invasions of *PB* transposons as young transposons have lower Kimura divergence than the older transposons [32].

## 4. Discussion

### 4.1. Distribution, Diversity, and Activity of PB in the Compact Genomes of Animals

With reference to the literature reviews, it can be observed that *PB* transposons are extensively dispersed and are found in most vertebrates, such as fishes, mammals and rodents, as well as various invertebrates including crustaceans, insects, nematodes, mollusks, corals, flatworms, etc. [33]. In this study, the distribution, diversity and activity of *PB* transposons in 79 compact genomes of animals were investigated. We observed that *PB* transposon types have been disclosed in approximately half (47%) of the total number of the species examined. These *PB* transposon types were irregularly distributed across various families of both vertebrate and invertebrate small genomes of animals. Comparatively, a significantly larger number of *PB* transposon types were noticed from *Aporrectodea caliginosa*, *Argiope trifasciata*, and *Girardia tigrina* than other species, as they contributed 28 (13%), 21 (9.9%), and 21 (9.9%), respectively, probably due to their high abundance. Though high diversity was observed among these *PB* transposon types, at least seven distinct clades of *PB* transposases were recognized, including the *Pokey* family, which was identified as a family of DNA transposon targeting rRNA genes [34]. The *PB* transposon types found in clade C and clade F are relatively conserved, and harbor high number of *PB* transposon types. Despite the significant range of *PB* transposons among the *PB* superfamily and *Tc1/mariner*, many other *PB* transposon essentials are also commonly found in *Arthropoda* [35]. We also found that the highest number of the *PB* transposon types mined from this study were detected from the phylum *Arthropoda*. Again, we identified seven different clades of *PB* transposases from both vertebrate and invertebrate genomes, with low sequence similarity among them. Our data provides evidence of a wide distribution, high diversity, and activity of *PB* transposons in compact genomes of animals.

During distribution analysis of the *PB* transposon types, we only considered a *PB* substance to be present in a given genome if at least one flanking transposon containing the terminal inverted repeat (TIR) and target site duplication (TSD) was detected. Any *PB*-derived sequences lacking detectable TSD and TIR, as well as domesticated genes, were excluded from the analysis. Additionally, we excluded *PB*-related miniature inverted-repeat transposable elements (MITEs), which have lost open reading frames (ORFs) [36], as they may exist in these genomes. This exclusion was implemented by conducting a local TBLASTN search with the DDE domain of the *PB* transposase in our mining protocol. However, it is possible that this exclusion resulted in the elimination of many *PB* elements that are truncated and have short lengths, leading to underestimation of the taxonomic distribution of *PB* transposons in genomes. Moreover, as a result of continuous rivalry in genome sequencing, analysis and updates, more novel families of *PB* transposons are likely to be uncovered from the *PB* superfamily with higher variable potentials than this study has perceived.

Domestication events have been observed in well-defined DNA transposons, such as the *pogo* superfamily which exhibit repeated domestications in vertebrates [37]. Recurrent domestications of *hAT* transposons in vertebrates were also observed, leading to the identification of at least 6 *ZBED* genes that contain Zinc-finger BED domains derived from *hAT* transposases [38]. Profiling the molecular evolution of *PB* transposons revealed the existence of at least eight domestication genes derived from *PB* transposases [39]. Since *PB* transposases are only detected in 37 species with small genomes, it is challenging to distinguish these sequences from truncated transposons, pseudogenes, or domesticated genes. Consequently, we excluded them from our analysis.

Again, for detailed enquiry, we made the *PB* transposon types discovered undergo several processes of amplification, diversification, inactivation and elimination stages. The intact *PB* copies obtained indicate a recent amplification of the *PB* transposons, suggesting that they may be active [40]. The total intact copy number of the *PB* transposon types were very low in most of the species, especially that of the Tetraodontiformes genome, suggesting that *PB* transposons do not show much amplification yet in the vertebrate compact genomes. However, some species, such as *Argiope trifasciata*, *Biomphalaria glabrata* and *Daphnia obtuse*, display highly intact *PB* copies. This signifies that the *PB* transposons in these genomes might be very young and still maintain transposition activities, which is worth further investigation. In summary, most of the *PB* transposon types found are short, truncated and lack efficient transposase domains, TIRs and transposition force of actions [41]. *PB* transposons are erratic and not common in the small genomes of animals because out of the 212 *PB* transposon types investigated, 188 *PB* transposon types recorded less than six intact *PB* copy numbers, representing 88.7%. Nevertheless, only 24 *PB* transposon types originating from eight species were discovered to harbor more than five intact copy numbers. This is contrarily to other DNA transposon families of *Tc1/mariner* that have been identified [42], indicating that there might be a specific unknown factor in the small genomes of animals, which restrict the expansion and display of DNA transposons to maintain small sizes as discovered.

### 4.2. Structural Organization of PiggyBac in the Animal Genome

This study acknowledged that most of the complete *PB* transposon naked sequences out from the compact animal genomes are similar to the original sequences harbored in *Trichoplusia ni*, the cabbage looper worm, as they consist of and display all five main domains that have ever been discussed [8]. Though various studies revealed that TIRs are very key to transposase recognition and target site cleavage with first two base pairs mutations resulting in excision [39], this study also showed that a large number of the *PB* transposons detected harbor the conserved and consistent CCC/CCC in their first three TIR base pairs, with few or no mutations. A few *PB* transposons, such as PiggyBac-1_Csap and PiggyBac-2_Lvar from *Callinectes sapidus* and *Lytechinus variegatus*, respectively, have very short identifiable TIRs (4 bp and 5 bp), indicating that they are truncated. Almost all the *PB* transposons obtained exhibited the TSDs of the conserved TTAA, with the exception of few transposons that display diverse TIRs of CTAA, ATTA, TCAA, TATG, TTAG and TAAA, suggesting the existence of very little probable mutations in action. This study discovered that all the clades harbor nearly the same characteristic domains. The catalytic domain (DDD) between and within the various clades had high similarities than that of NTD. The distance between the second and third aspartate residues of the DDD domains among the seven clades vary, making them not conserved as perceived. Our data is consistent with previous findings, emphasizing the presence of five major domains in *PB* transposases: the N-terminal domain (NTD), Dimerization and DNA-binding domain 1 (DDBD1), Catalytic domain (either DDD/DDE), Dimerization and DNA-binding domain 2 (DDBD2), and the C-terminal cysteine-rich domain (CRD) [8]. It is important to note that the CRD, DDBD1, DDBD2 and NTD domains show lower conservation, with low sequence identities observed within and between the clades. Particularly, the N-terminal domain (NTD) displays very limited sequence similarity across most clades (<0.1). The NTD domain, responsible for TIRs binding, exhibits the lowest level of preservation among all investigated domains, but shares similarities with other transposases in a highly diverse manner. On the other hand, the DDD domain, responsible for catalyzing the transposition reaction of the transposons, is highly conserved among the transposases of all seven examined clades [43].

## 5. Conclusions

This study provides comprehensive data on the distribution and diversity of *PB* transposon types in 79 species of small genomes of animals, along with an analysis of their activity, phylogeny and structural characteristics. In summary, *PB* transposons are widely distributed across both vertebrate and invertebrate small genomes, and can be categorized into seven clades based on IQ-Tree analysis. Though low sequence similarities were recorded among the various clades examined, each individual clade displays and shows that the DDD domains they harbor are highly unique and conserved.

Again, in spite of the fact that our data shows low copy number and intact copy number in most *PB* transposon types among the small genomes of 37 species, the evolution dynamics and sequence analysis of the discovered *PB* transposon types revealed that *PB* transposons may be active in some small genomes of animals.

## Figures and Tables

**Table 1:** *PB* transposons in small genomes of animals.

Taxa Distribution	Order	Species	Number of Species Inquired	Number of Species Containing *PB*	Number of Types Containing FL *PB*	Number of Types Containing Intact *PB* Copies	Length of the FL *PB* ^a^	Length of the Intact *PB* ^b^	Transposase Length of the Intact *PB*	TIR Length of the Intact *PB*
Annelida			7	6						
	Crassiclitellata	*Aporrectodea caliginosa*			28	14	1416–5657	2223–5657	507–1316	8–61
		*Eisenia fetida*			1	-	2167	-	-	-
		*Lumbricus rubellus*			11	7	1705–4295	2318–4295	574–925	13–18
	Rhynchobdellida	*Helobdella robusta*			6	4	2190–2724	2313–2724	535–587	12–15
	Hirudinida	*Hirudo medicinalis*			1	-	1483–1518	-	-	-
	Terebellida	*Paralvinella palmiformis*			1	1	2587	2587	503	13
Arthropoda			30	13						
	Araneae	*Argiope aurantia*			16	3	1765–9505	2314–9505	544–577	13–18
		*Argiope trifasciata*			21	8	1291–7726	2125–7726	500–640	12–18
		*Latrodectus hesperus*			7	1	1773–3135	2168	558	6
		*Loxosceles reclusa*			2	-	1314–2035	-	-	-
		*Parasteatoda tepidariorum*			5	1	1680–2903	2133	564	7–16
		*Tetragnatha versicolor*			7	2	1673–5404	3717–5404	584–687	9–14
	Balanomorpha	*Amphibalanus amphitrite*			5	3	1965–4732	2708–4732	506–645	13–18
	Decapoda	*Callinectes sapidus*			4	2	2177–4527	2763–3434	554–629	13–24
	Diplostraca	*Daphnia obtusa*			2	2	5295–6986	5295–6986	506–714	12–14
		*Daphnia pulex*			1	-	2607–4619	-	-	-
		*Daphnia pulicaria*			2	2	4407–8943	4407–8943	554–921	14–16
	Sarcoptiformes	*Archegozetes longisetosus*			2	2	2348–2362	2348–2359	523–567	7–11
	Siphonostomatoida	*Lepeophtheirus salmonis*			2	-	1698–1739	-	-	-
Chordata			10	6						
	Tetraodontiformes	*Mola mola*			1	-	2335–2350	-	-	-
		*Pao palembangensis*			2	1	1321–3029	3029	548	14
		*Takifugu bimaculatus*			4	2	1234–3869	3386–3869	549–562	14–35
		*Takifugu flavidus*			3	-	1966–3373	-	-	-
		*Takifugu ocellatus*			2	-	1384–2056	-	-	-
		*Takifugu rubripes*			3	1	1967–2750	2750	558	14
Echinodermata			6	2						
	Temnopleuroida	*Lytechinus pictus*			1	1	4650	4650	560	16
		*Lytechinus variegatus*			2	2	4777–5974	4777–5974	559–614	5–16
Mollusca			10	8						
	Architaenioglossa	*Biomphalaria glabrata*			9	9	2226–3072	2226–3072	505–675	5–17
	Chitonida	*Acanthopleura granulata*			2	2	2982–3061	2982–3061	522–703	15
	Mytilida	*Mytilisepta virgata*			6	1	2433–5661	4089–4133	543	16
	Ostreida	*Crassostrea gigas*			4	2	2348–6085	2348–2590	507–595	14–16
		*Crassostrea virginica*			6	1	3850–6572	5268–5289	523–586	9
		*Ostrea edulis*			4	3	2347–3184	2347–2678	507–638	12–14
	Pectinida	*Argopecten irradians irradians*			4	1	3130–4412	4047–4053	590–626	15
		*Pinctada fucata*			1	-	5595	-	-	-
Platyhelminthes			6	2						
	Tricladida	*Girardia tigrina*			21	1	1265–3478	2624–2627	504–525	13
		*Schmidtea mediterranea*			13	6	2126–3184	2359–3184	509–652	13–17

^a^ FL *PB*: Transposons flanked by detectable target site duplications (TSDs) and TIRs; ^b^ intact *PB*: Transposons flanked by detectable TSDs and TIRs and encoded ≥500 aa transposases.

## Data Availability

Data are contained within the article and Supplementary Materials.

## Supplementary Materials

The following supporting information can be downloaded at: https://www.mdpi.com/article/10.3390/biology13010024/s1, Figure S1: Relationship between genome size and *piggyBac* Transposon’s diversity, abundance, and activity; Figure S2: Complete evolutionary tree; Figure S3: Multiple sequence alignments of *piggyBac* domains; Table S1: Animal genomes information; Table S2: 212 different types of *piggyBac* transposons information.
