# Supplementary material for: PiggyBac Transposon Mining in the Small Genomes of Animals
Source: Biology (Basel). 2023 Dec 31;13(1):24. doi: 10.3390/biology13010024 (PMC10813416; doi:10.3390/biology13010024)
Supplement: Supplementary file 1 [file biology-13-00024-s001.zip › Supplement/Figure S3.pdf]

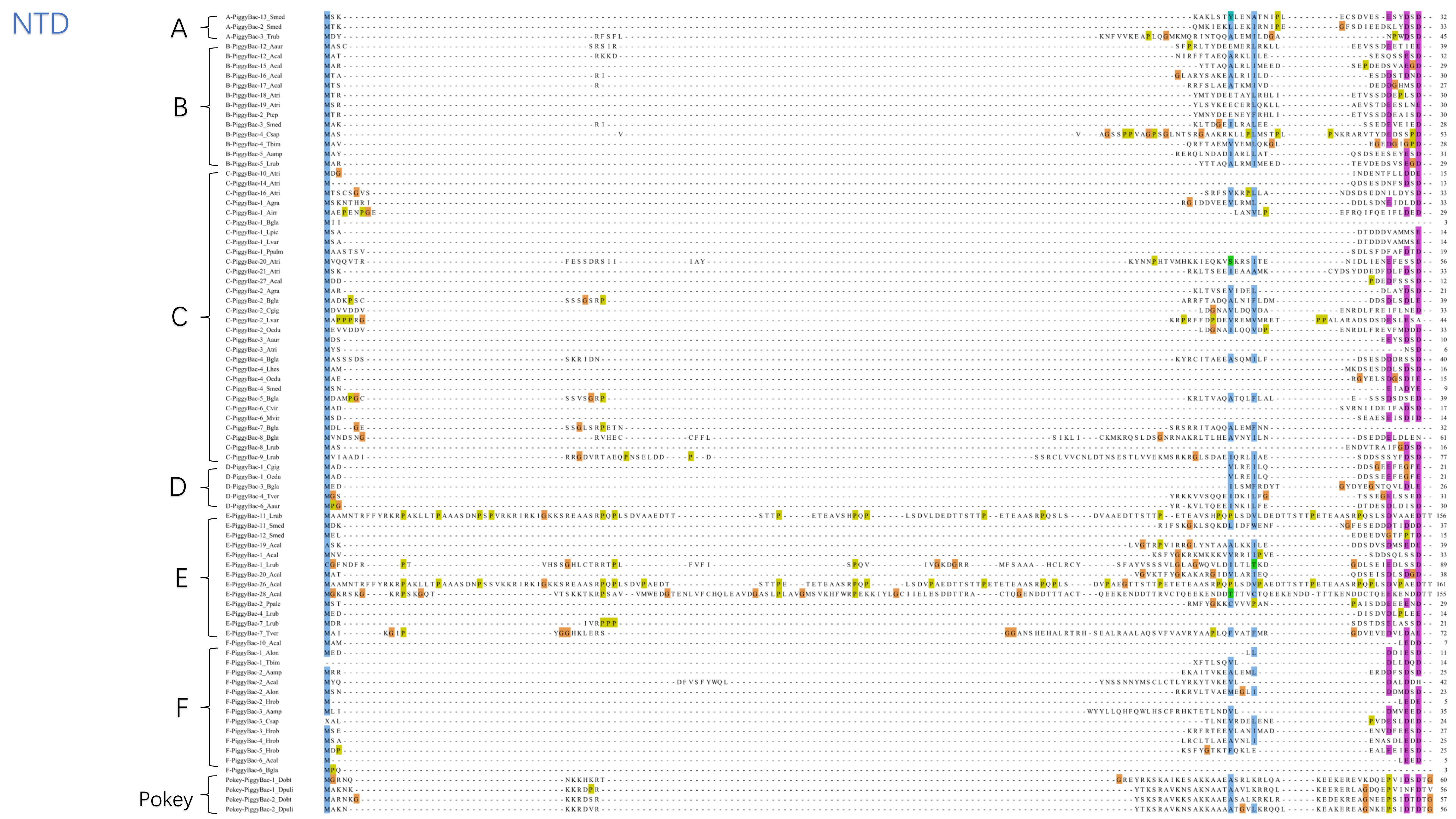
NTD
A
A-PiggyBac-13_Smed
A-PiggyBac-2_Smed
A-PiggyBac-3_Trub
B
B-PiggyBac-12_Aaur
B-PiggyBac-12_Acal
B-PiggyBac-15_Acal
B-PiggyBac-16_Acal
B-PiggyBac-17_Acal
B-PiggyBac-18_Atri
B-PiggyBac-19_Atri
B-PiggyBac-2_Ptep
B-PiggyBac-3_Smed
B-PiggyBac-4_Csap
B-PiggyBac-4_Tbim
B-PiggyBac-5_Aamp
B-PiggyBac-5_Lrub
C
C-PiggyBac-10_Atri
C-PiggyBac-14_Atri
C-PiggyBac-16_Atri
C-PiggyBac-1_Agra
C-PiggyBac-1_Airr
C-PiggyBac-1_Bgla
C-PiggyBac-1_Lpic
C-PiggyBac-1_Lvar
C-PiggyBac-1_Ppalm
C-PiggyBac-20_Atri
C-PiggyBac-21_Atri
C-PiggyBac-27_Acal
C-PiggyBac-2_Agra
C-PiggyBac-2_Bgla
C-PiggyBac-2_Cgig
C-PiggyBac-2_Lvar
C-PiggyBac-2_Oedu
C-PiggyBac-3_Aaur
C-PiggyBac-3_Atri
C-PiggyBac-4_Bgla
C-PiggyBac-4_Lhes
C-PiggyBac-4_Oedu
C-PiggyBac-4_Smed
C-PiggyBac-5_Bgla
C-PiggyBac-6_Cvir
C-PiggyBac-6_Mvir
C-PiggyBac-7_Bgla
C-PiggyBac-8_Bgla
C-PiggyBac-8_Lrub
C-PiggyBac-9_Lrub
D
D-PiggyBac-1_Cgig
D-PiggyBac-1_Oedu
D-PiggyBac-3_Bgla
D-PiggyBac-4_Tver
D-PiggyBac-6_Aaur
E
E-PiggyBac-11_Lrub
E-PiggyBac-11_Smed
E-PiggyBac-12_Smed
E-PiggyBac-19_Acal
E-PiggyBac-1_Acal
E-PiggyBac-1_Lrub
E-PiggyBac-20_Acal
E-PiggyBac-26_Acal
E-PiggyBac-28_Acal
E-PiggyBac-2_Ppale
E-PiggyBac-4_Lrub
E-PiggyBac-7_Lrub
E-PiggyBac-7_Tver
F
F-PiggyBac-10_Acal
F-PiggyBac-1_Alon
F-PiggyBac-1_Tbim
F-PiggyBac-2_Aamp
F-PiggyBac-2_Acal
F-PiggyBac-2_Alon
F-PiggyBac-2_Hrob
F-PiggyBac-3_Aamp
F-PiggyBac-3_Csap
F-PiggyBac-3_Hrob
F-PiggyBac-4_Hrob
F-PiggyBac-5_Hrob
F-PiggyBac-6_Acal
F-PiggyBac-6_Bgla
Pokey
Pokey-PiggyBac-1_Dobt
Pokey-PiggyBac-1_Dpuli
Pokey-PiggyBac-2_Dobt
Pokey-PiggyBac-2_Dpuli

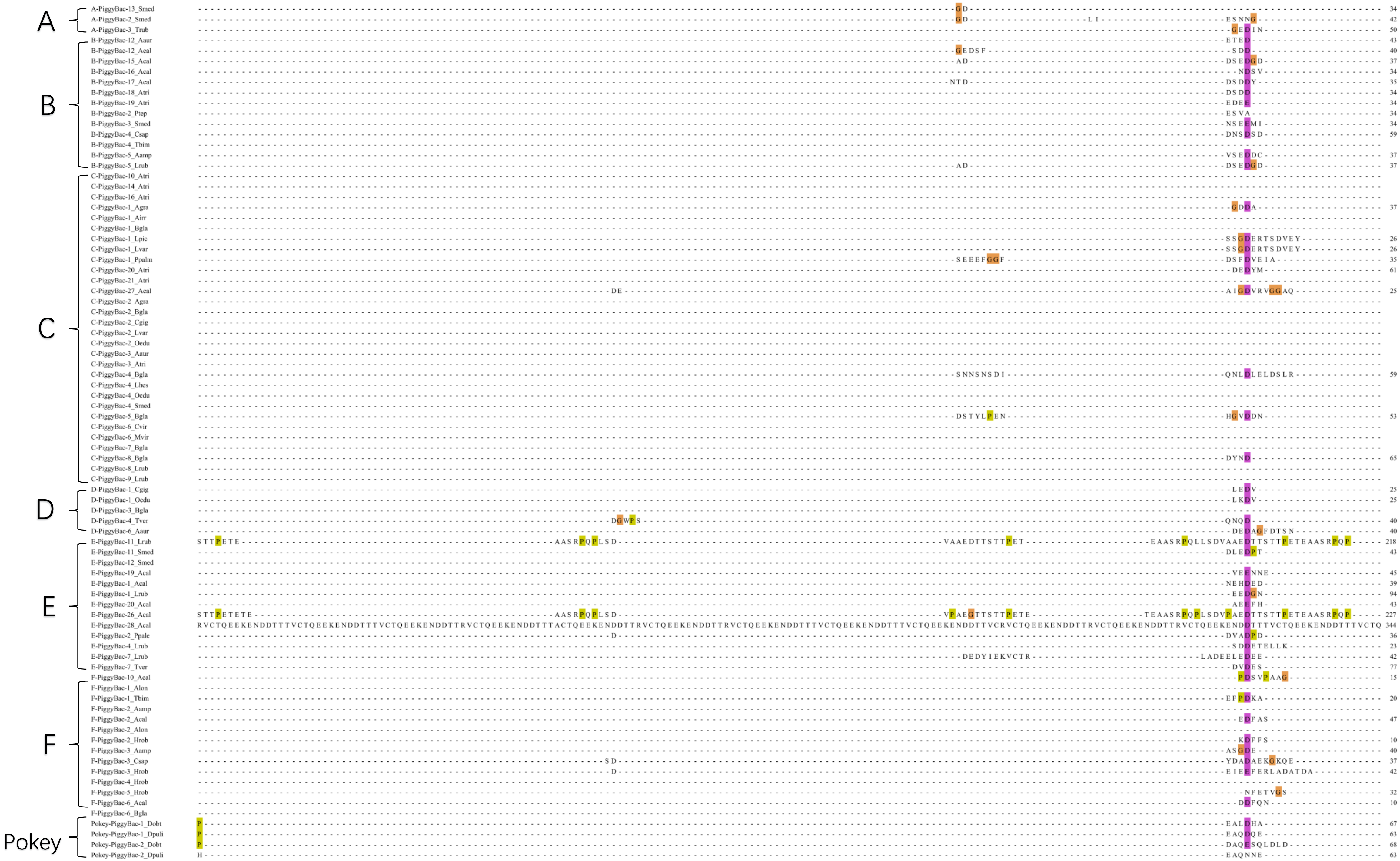
A
B
C
D
E
F
Pokey

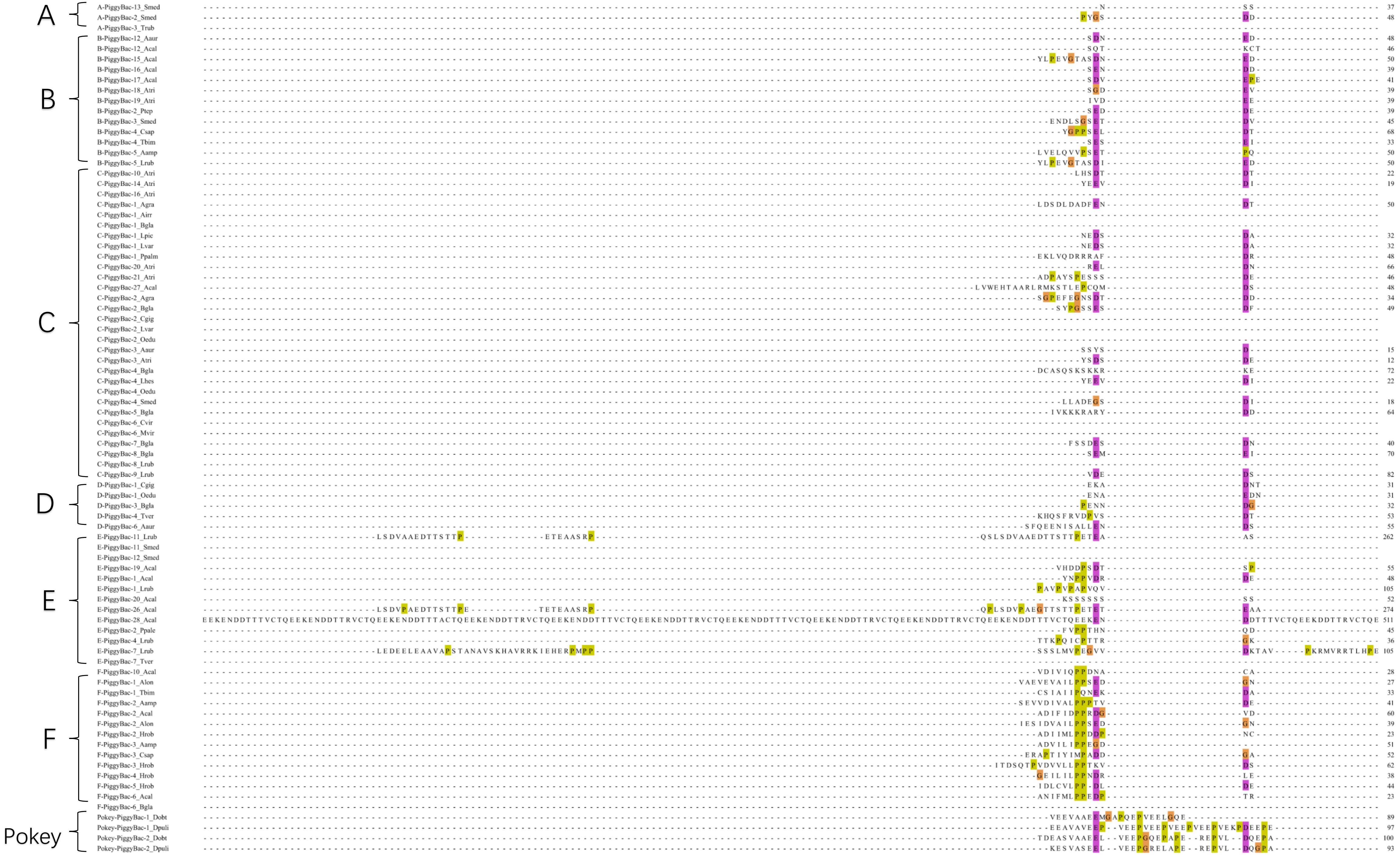
A
A-PiggyBac-13_Smed
A-PiggyBac-2_Smed
A-PiggyBac-3_Trub
B
B-PiggyBac-12_Aaur
B-PiggyBac-12_Acal
B-PiggyBac-15_Acal
B-PiggyBac-16_Acal
B-PiggyBac-17_Acal
B-PiggyBac-18_Atri
B-PiggyBac-19_Atri
B-PiggyBac-2_Ptep
B-PiggyBac-3_Smed
B-PiggyBac-4_Csap
B-PiggyBac-4_Tbim
B-PiggyBac-5_Aamp
B-PiggyBac-5_Lrub
C
C-PiggyBac-10_Atri
C-PiggyBac-14_Atri
C-PiggyBac-16_Atri
C-PiggyBac-1_Agra
C-PiggyBac-1_Airr
C-PiggyBac-1_Bgla
C-PiggyBac-1_Lpic
C-PiggyBac-1_Lvar
C-PiggyBac-1_Ppalm
C-PiggyBac-20_Atri
C-PiggyBac-21_Atri
C-PiggyBac-27_Acal
C-PiggyBac-2_Agra
C-PiggyBac-2_Bgla
C-PiggyBac-2_Cgig
C-PiggyBac-2_Lvar
C-PiggyBac-2_Oedu
C-PiggyBac-3_Aaur
C-PiggyBac-3_Atri
C-PiggyBac-4_Bgla
C-PiggyBac-4_Lhes
C-PiggyBac-4_Oedu
C-PiggyBac-4_Smed
C-PiggyBac-5_Bgla
C-PiggyBac-6_Cvir
C-PiggyBac-6_Mvir
C-PiggyBac-7_Bgla
C-PiggyBac-8_Bgla
C-PiggyBac-8_Lrub
C-PiggyBac-9_Lrub
D
D-PiggyBac-1_Cgig
D-PiggyBac-1_Oedu
D-PiggyBac-3_Bgla
D-PiggyBac-4_Tver
D-PiggyBac-6_Aaur
E
E-PiggyBac-11_Lrub
E-PiggyBac-11_Smed
E-PiggyBac-12_Smed
E-PiggyBac-19_Acal
E-PiggyBac-1_Acal
E-PiggyBac-1_Lrub
E-PiggyBac-20_Acal
E-PiggyBac-26_Acal
E-PiggyBac-28_Acal
E-PiggyBac-2_Ppale
E-PiggyBac-4_Lrub
E-PiggyBac-7_Lrub
E-PiggyBac-7_Tver
F
F-PiggyBac-10_Acal
F-PiggyBac-1_Alon
F-PiggyBac-1_Tbim
F-PiggyBac-2_Aamp
F-PiggyBac-2_Acal
F-PiggyBac-2_Alon
F-PiggyBac-2_Hrob
F-PiggyBac-3_Aamp
F-PiggyBac-3_Csap
F-PiggyBac-3_Hrob
F-PiggyBac-4_Hrob
F-PiggyBac-5_Hrob
F-PiggyBac-6_Acal
F-PiggyBac-6_Bgla
Pokey
Pokey-PiggyBac-1_Dobt
Pokey-PiggyBac-1_Dpuli
Pokey-PiggyBac-2_Dobt
Pokey-PiggyBac-2_Dpuli

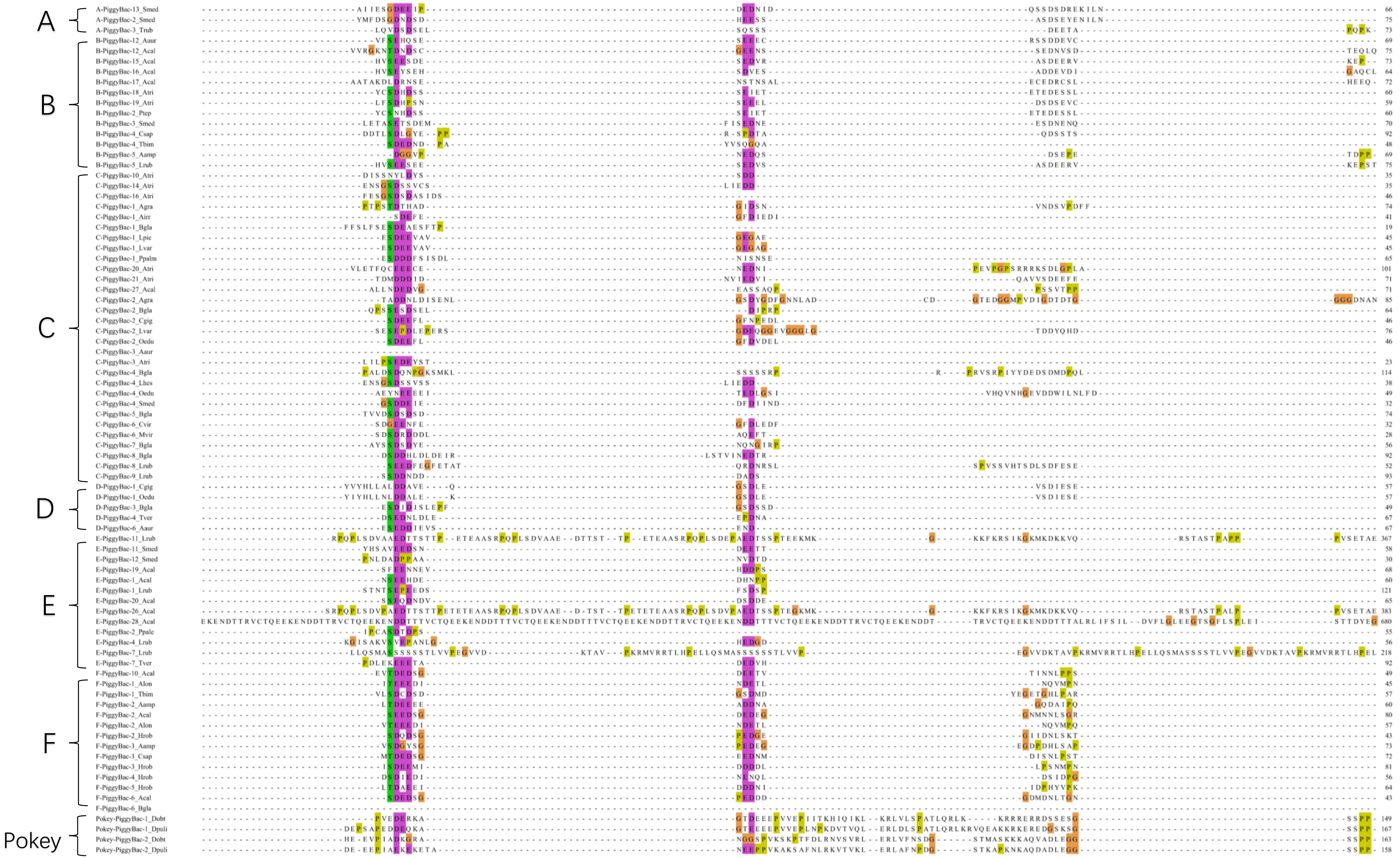
A
A-PiggyBac-13_Smed
A-PiggyBac-2_Smed
A-PiggyBac-3_Trub
B
B-PiggyBac-12_Aaur
B-PiggyBac-12_Acal
B-PiggyBac-15_Acal
B-PiggyBac-16_Acal
B-PiggyBac-17_Acal
B-PiggyBac-18_Atri
B-PiggyBac-19_Atri
B-PiggyBac-2_Ptep
B-PiggyBac-3_Smed
B-PiggyBac-4_Csap
B-PiggyBac-4_Tbim
B-PiggyBac-5_Aamp
B-PiggyBac-5_Lrub
C
C-PiggyBac-10_Atri
C-PiggyBac-14_Atri
C-PiggyBac-16_Atri
C-PiggyBac-1_Agra
C-PiggyBac-1_Airr
C-PiggyBac-1_Bgla
C-PiggyBac-1_Lpic
C-PiggyBac-1_Lvar
C-PiggyBac-1_Ppalm
C-PiggyBac-20_Atri
C-PiggyBac-21_Atri
C-PiggyBac-27_Acal
C-PiggyBac-2_Agra
C-PiggyBac-2_Bgla
C-PiggyBac-2_Cgig
C-PiggyBac-2_Lvar
C-PiggyBac-2_Oedu
C-PiggyBac-3_Aaur
C-PiggyBac-3_Atri
C-PiggyBac-4_Bgla
C-PiggyBac-4_Lhes
C-PiggyBac-4_Oedu
C-PiggyBac-4_Smed
C-PiggyBac-5_Bgla
C-PiggyBac-6_Cvir
C-PiggyBac-6_Mvir
C-PiggyBac-7_Bgla
C-PiggyBac-8_Bgla
C-PiggyBac-8_Lrub
C-PiggyBac-9_Lrub
D
D-PiggyBac-1_Cgig
D-PiggyBac-1_Oedu
D-PiggyBac-3_Bgla
D-PiggyBac-4_Tver
D-PiggyBac-6_Aaur
E
E-PiggyBac-11_Lrub
E-PiggyBac-11_Smed
E-PiggyBac-12_Smed
E-PiggyBac-19_Acal
E-PiggyBac-1_Acal
E-PiggyBac-1_Lrub
E-PiggyBac-20_Acal
E-PiggyBac-26_Acal
E-PiggyBac-28_Acal
E-PiggyBac-2_Ppale
E-PiggyBac-4_Lrub
E-PiggyBac-7_Lrub
E-PiggyBac-7_Tver
F
F-PiggyBac-10_Acal
F-PiggyBac-1_Alon
F-PiggyBac-1_Tbim
F-PiggyBac-2_Aamp
F-PiggyBac-2_Acal
F-PiggyBac-2_Alon
F-PiggyBac-2_Hrob
F-PiggyBac-3_Aamp
F-PiggyBac-3_Csap
F-PiggyBac-3_Hrob
F-PiggyBac-4_Hrob
F-PiggyBac-5_Hrob
F-PiggyBac-6_Acal
F-PiggyBac-6_Bgla
Pokey
Pokey-PiggyBac-1_Dobt
Pokey-PiggyBac-1_Dpuli
Pokey-PiggyBac-2_Dobt
Pokey-PiggyBac-2_Dpuli

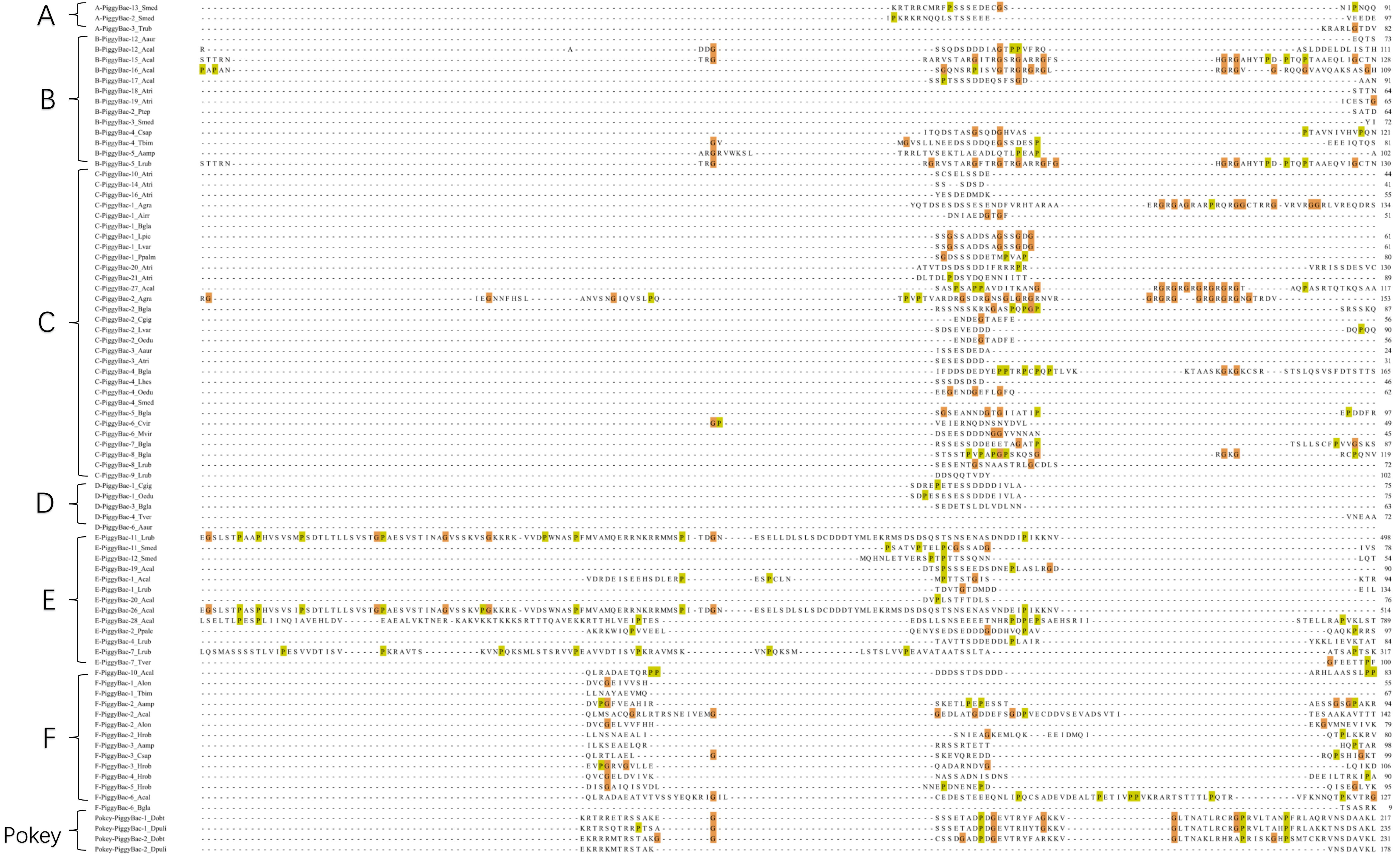
A
A-PiggyBac-13_Smed
A-PiggyBac-2_Smed
A-PiggyBac-3_Trub
B
B-PiggyBac-12_Aaur
B-PiggyBac-12_Acal
B-PiggyBac-15_Acal
B-PiggyBac-16_Acal
B-PiggyBac-17_Acal
B-PiggyBac-18_Atri
B-PiggyBac-19_Atri
B-PiggyBac-2_Ptep
B-PiggyBac-3_Smed
B-PiggyBac-4_Csap
B-PiggyBac-4_Tbim
B-PiggyBac-5_Aamp
B-PiggyBac-5_Lrub
C
C-PiggyBac-10_Atri
C-PiggyBac-14_Atri
C-PiggyBac-16_Atri
C-PiggyBac-1_Agra
C-PiggyBac-1_Airr
C-PiggyBac-1_Bgla
C-PiggyBac-1_Lpic
C-PiggyBac-1_Lvar
C-PiggyBac-1_Ppalm
C-PiggyBac-20_Atri
C-PiggyBac-21_Atri
C-PiggyBac-27_Acal
C-PiggyBac-2_Agra
C-PiggyBac-2_Bgla
C-PiggyBac-2_Cgig
C-PiggyBac-2_Lvar
C-PiggyBac-2_Oedu
C-PiggyBac-3_Aaur
C-PiggyBac-3_Atri
C-PiggyBac-4_Bgla
C-PiggyBac-4_Lhes
C-PiggyBac-4_Oedu
C-PiggyBac-4_Smed
C-PiggyBac-5_Bgla
C-PiggyBac-6_Cvir
C-PiggyBac-6_Mvir
C-PiggyBac-7_Bgla
C-PiggyBac-8_Bgla
C-PiggyBac-8_Lrub
C-PiggyBac-9_Lrub
D
D-PiggyBac-1_Cgig
D-PiggyBac-1_Oedu
D-PiggyBac-3_Bgla
D-PiggyBac-4_Tver
D-PiggyBac-6_Aaur
E
E-PiggyBac-11_Lrub
E-PiggyBac-11_Smed
E-PiggyBac-12_Smed
E-PiggyBac-19_Acal
E-PiggyBac-1_Acal
E-PiggyBac-1_Lrub
E-PiggyBac-20_Acal
E-PiggyBac-26_Acal
E-PiggyBac-28_Acal
E-PiggyBac-2_Ppalc
E-PiggyBac-4_Lrub
E-PiggyBac-7_Lrub
E-PiggyBac-7_Tver
F
F-PiggyBac-10_Acal
F-PiggyBac-1_Alon
F-PiggyBac-1_Tbim
F-PiggyBac-2_Aamp
F-PiggyBac-2_Acal
F-PiggyBac-2_Alon
F-PiggyBac-2_Hrob
F-PiggyBac-3_Aamp
F-PiggyBac-3_Csap
F-PiggyBac-3_Hrob
F-PiggyBac-4_Hrob
F-PiggyBac-5_Hrob
F-PiggyBac-6_Acal
F-PiggyBac-6_Bgla
Pokey
Pokey-PiggyBac-1_Dobt
Pokey-PiggyBac-1_Dpuli
Pokey-PiggyBac-2_Dobt
Pokey-PiggyBac-2_Dpuli

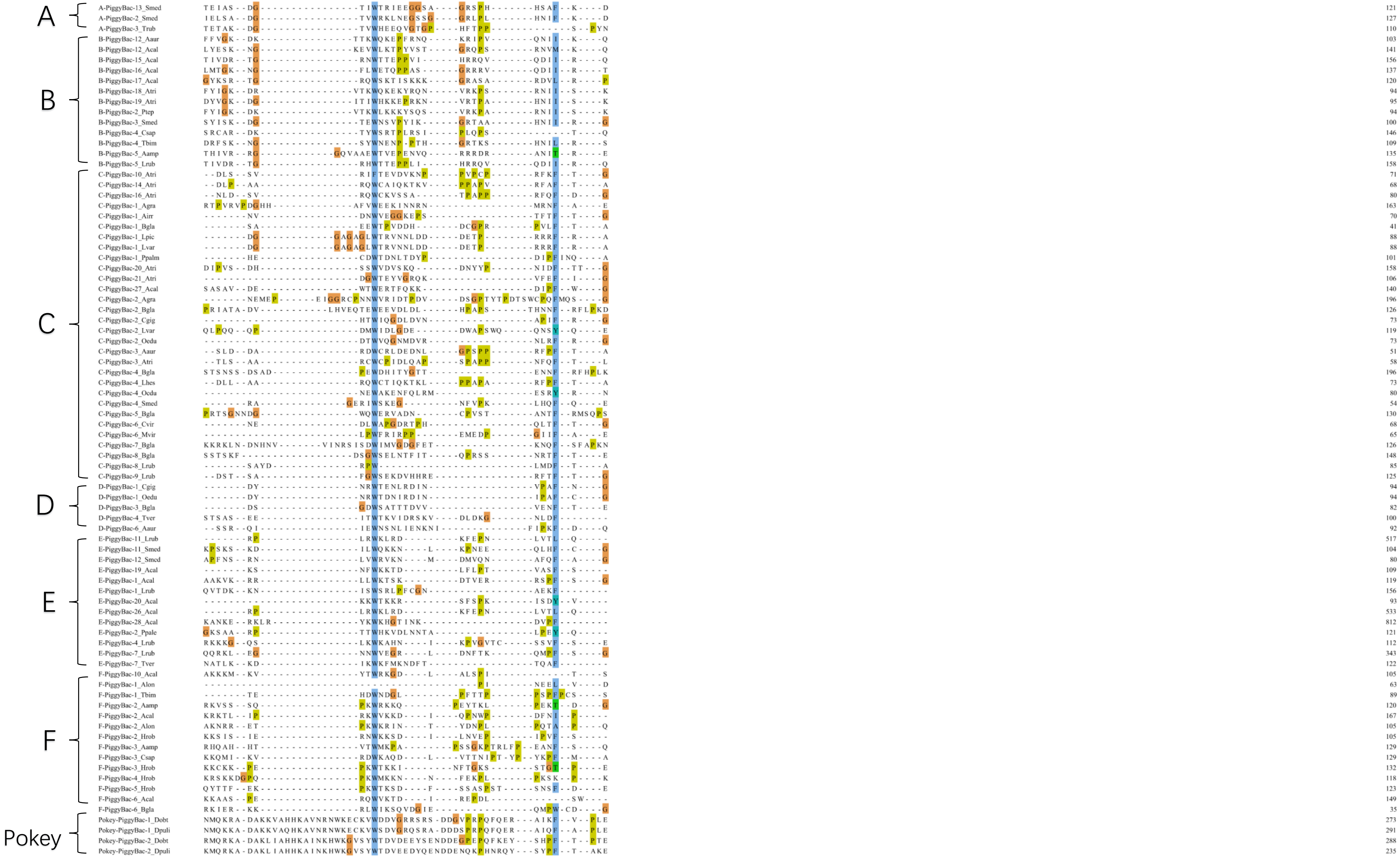
A
B
C
D
E
F
Pokey

DDBD1

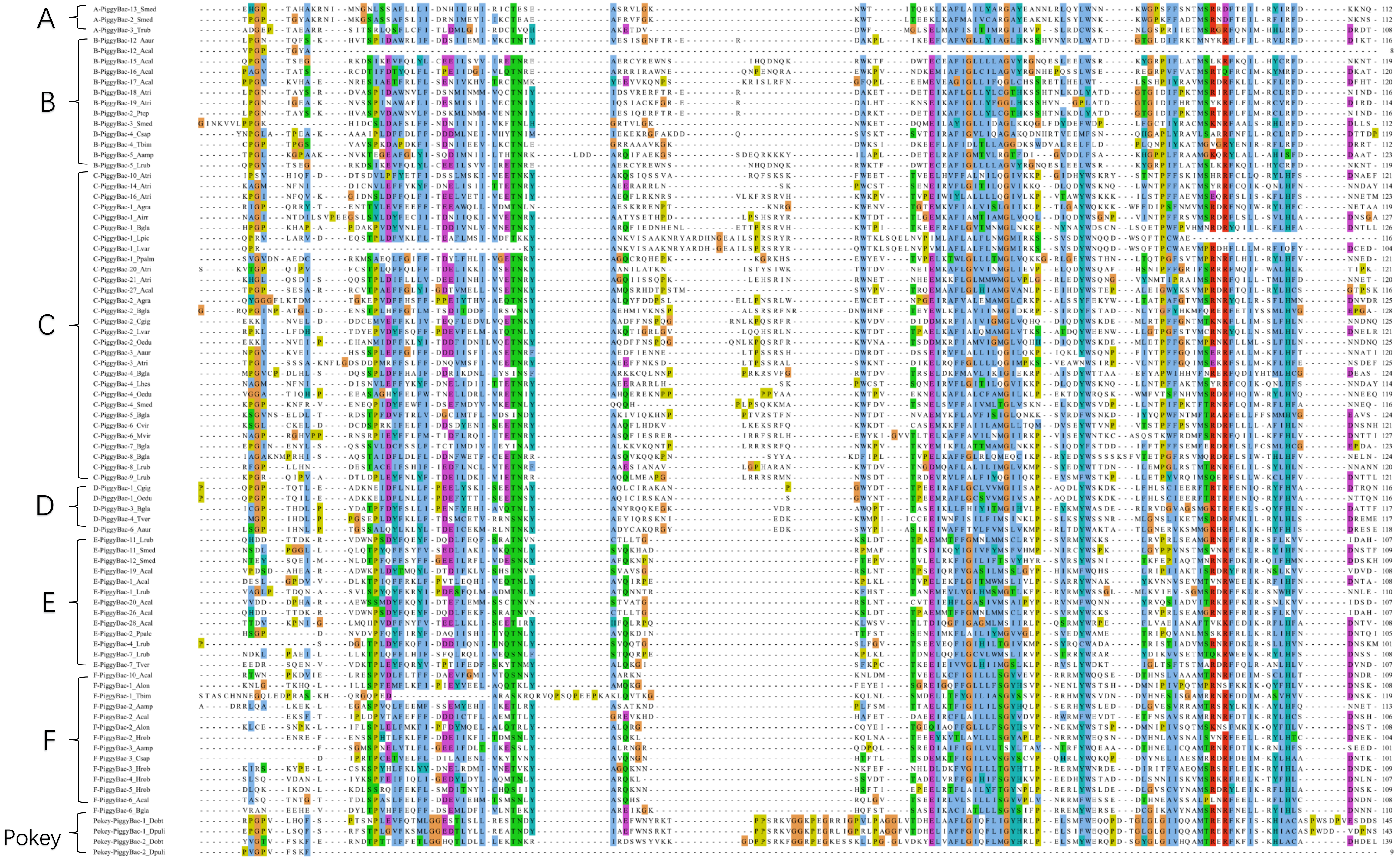

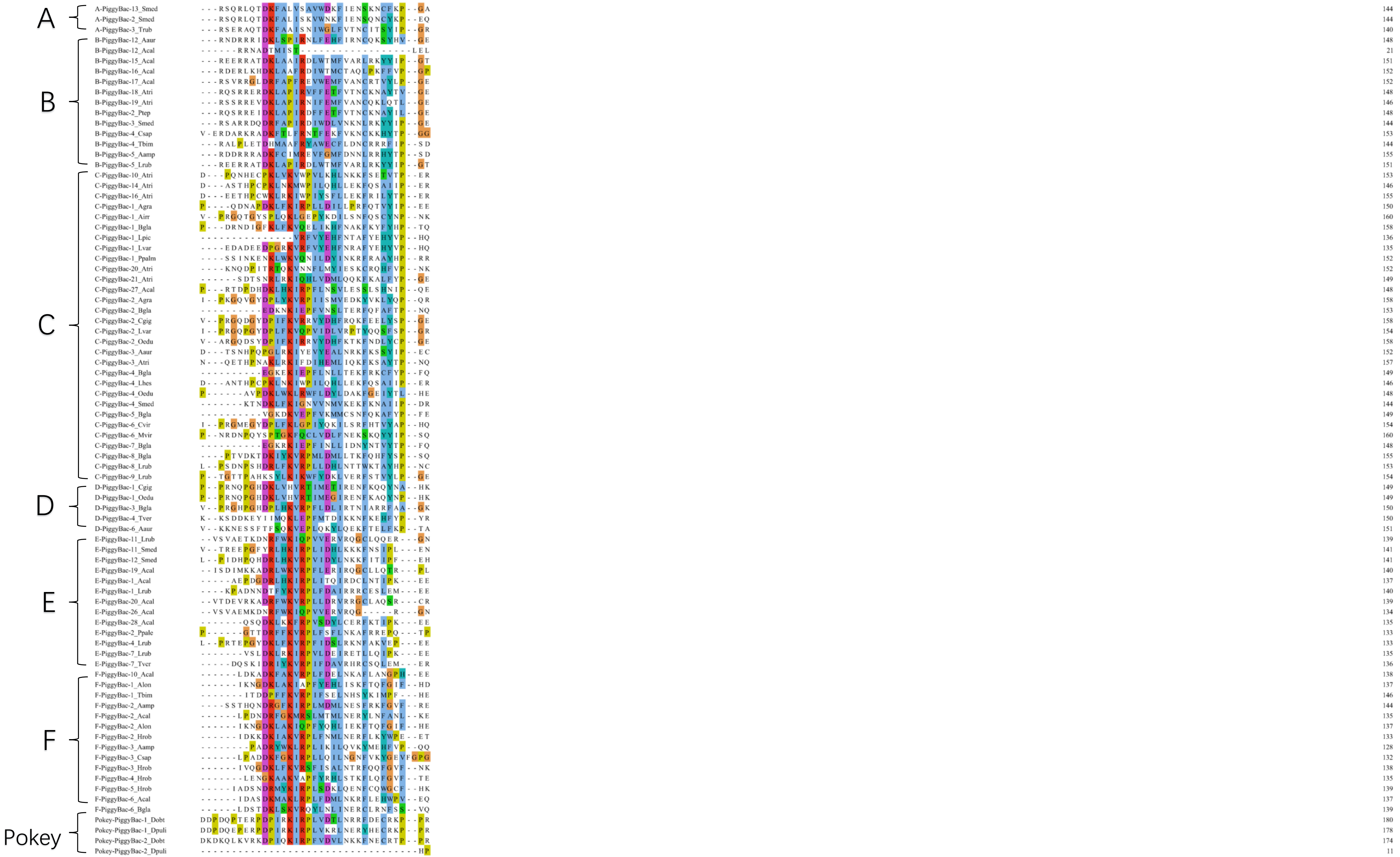

A
B
C
D
E
F
Pokey

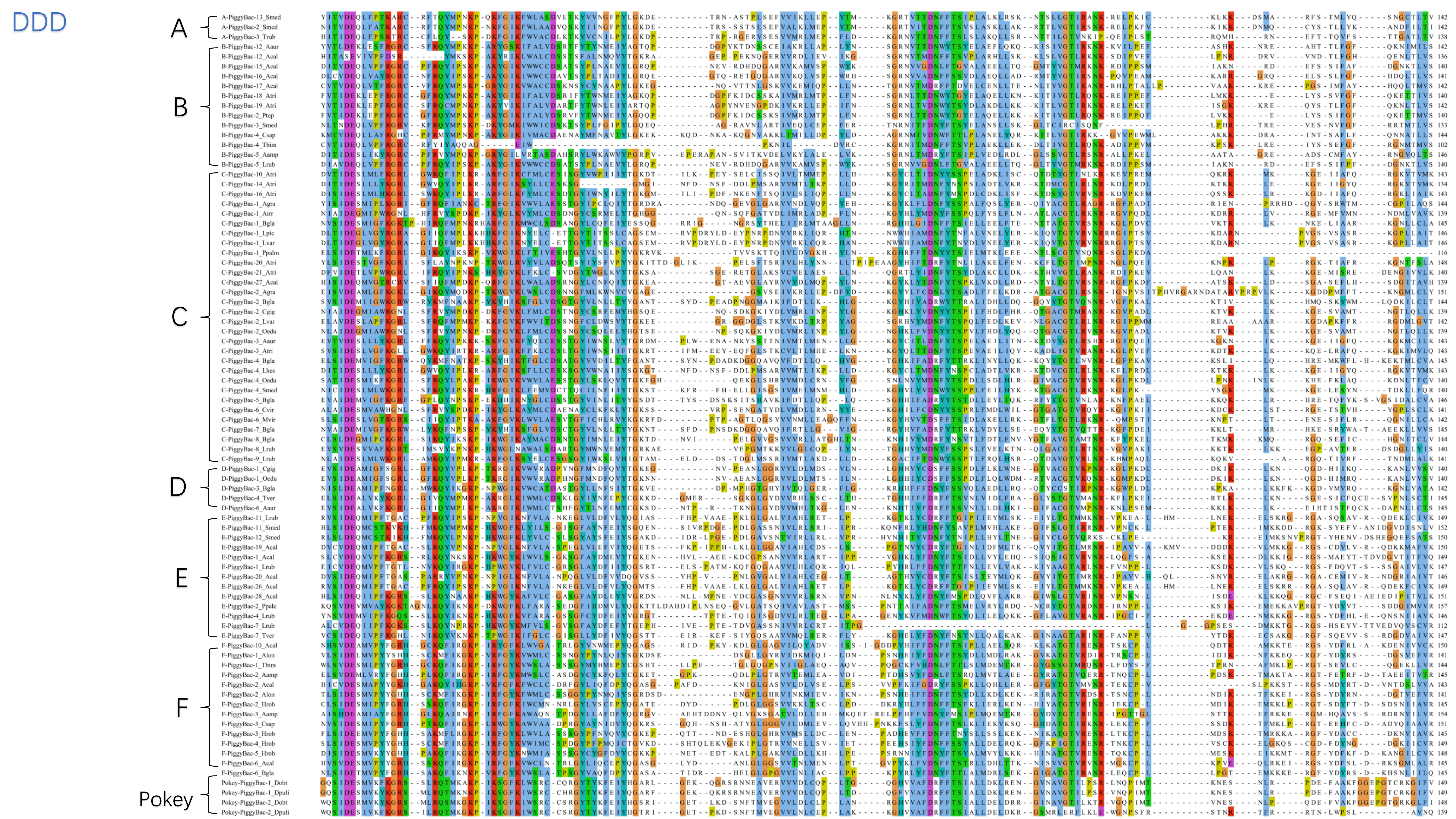

DDD
A
B
C
D
E
F
Pokey

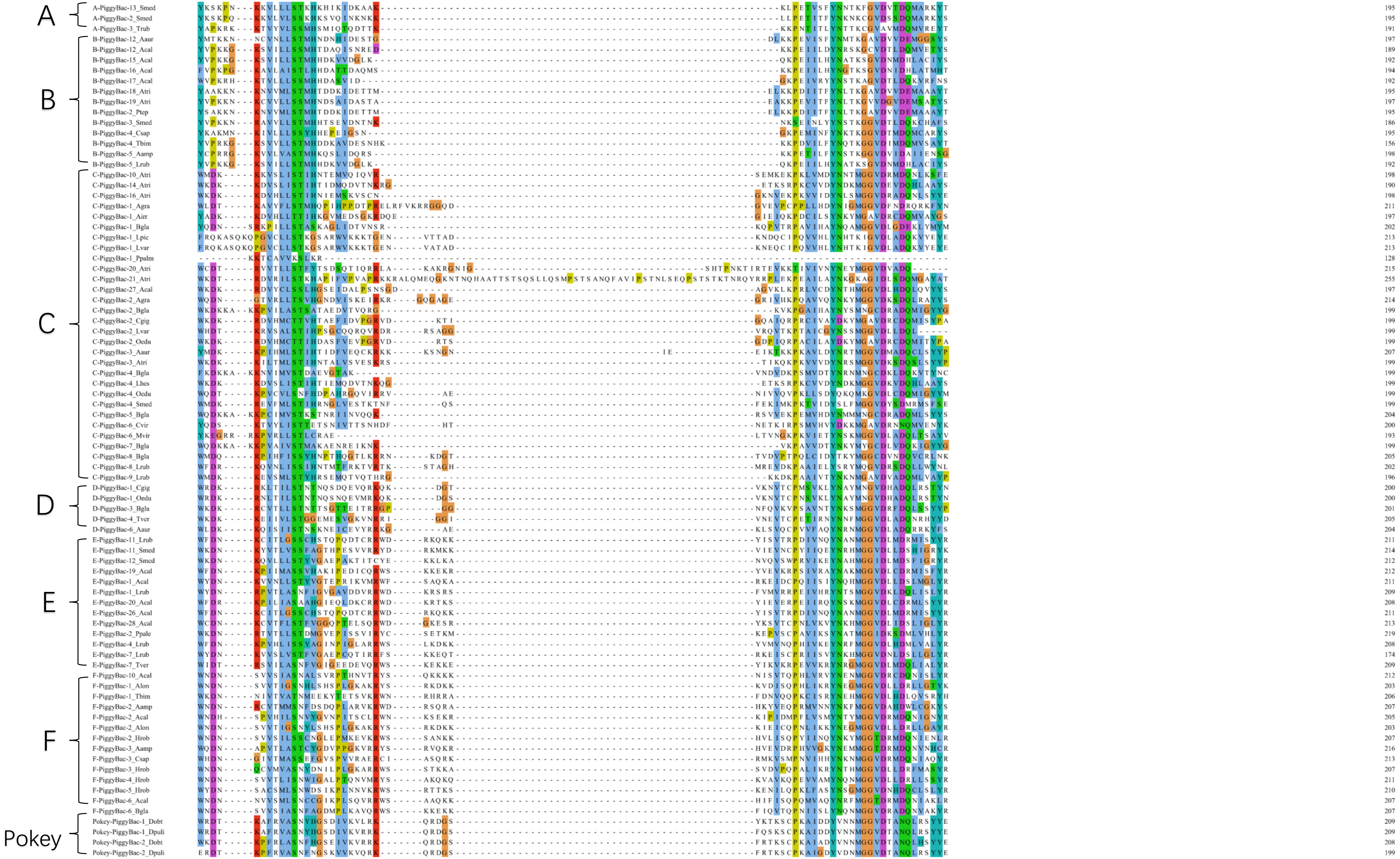
A
A-PiggyBac-13_Smed 195
A-PiggyBac-2_Smed 195
A-PiggyBac-3_Trub 191
B
B-PiggyBac-12_Aaur 197
B-PiggyBac-12_Acal 189
B-PiggyBac-15_Acal 192
B-PiggyBac-16_Acal 194
B-PiggyBac-17_Acal 192
B-PiggyBac-18_Atri 195
B-PiggyBac-19_Atri 197
B-PiggyBac-2_Ptep 195
B-PiggyBac-3_Smed 186
B-PiggyBac-4_Csap 195
B-PiggyBac-4_Tbim 156
B-PiggyBac-5_Aamp 198
B-PiggyBac-5_Lrub 192
C
C-PiggyBac-10_Atri 198
C-PiggyBac-14_Atri 190
C-PiggyBac-16_Atri 198
C-PiggyBac-1_Agra 211
C-PiggyBac-1_Airr 197
C-PiggyBac-1_Bgla 202
C-PiggyBac-1_Lpic 213
C-PiggyBac-1_Lvar 213
C-PiggyBac-1_Ppalm 128
C-PiggyBac-20_Atri 215
C-PiggyBac-21_Atri 255
C-PiggyBac-27_Acal 197
C-PiggyBac-2_Agra 214
C-PiggyBac-2_Bgla 199
C-PiggyBac-2_Cgig 199
C-PiggyBac-2_Lvar 199
C-PiggyBac-2_Oedu 199
C-PiggyBac-3_Aaur 207
C-PiggyBac-3_Atri 199
C-PiggyBac-4_Bgla 199
C-PiggyBac-4_Lhes 199
C-PiggyBac-4_Oedu 199
C-PiggyBac-4_Smed 199
C-PiggyBac-5_Bgla 204
C-PiggyBac-6_Cvir 200
C-PiggyBac-6_Mvir 193
C-PiggyBac-7_Bgla 199
C-PiggyBac-8_Bgla 205
C-PiggyBac-8_Lrub 202
C-PiggyBac-9_Lrub 196
D
D-PiggyBac-1_Cgig 200
D-PiggyBac-1_Oedu 200
D-PiggyBac-3_Bgla 201
D-PiggyBac-4_Tver 205
D-PiggyBac-6_Aaur 204
E
E-PiggyBac-11_Lrub 211
E-PiggyBac-11_Smed 214
E-PiggyBac-12_Smed 212
E-PiggyBac-19_Acal 212
E-PiggyBac-1_Acal 211
E-PiggyBac-1_Lrub 209
E-PiggyBac-20_Acal 208
E-PiggyBac-26_Acal 211
E-PiggyBac-28_Acal 213
E-PiggyBac-2_Ppale 219
E-PiggyBac-4_Lrub 208
E-PiggyBac-7_Lrub 174
E-PiggyBac-7_Tver 209
F
F-PiggyBac-10_Acal 212
F-PiggyBac-1_Alon 203
F-PiggyBac-1_Tbim 206
F-PiggyBac-2_Aamp 207
F-PiggyBac-2_Acal 205
F-PiggyBac-2_Alon 203
F-PiggyBac-2_Hrob 207
F-PiggyBac-3_Aamp 216
F-PiggyBac-3_Csap 213
F-PiggyBac-3_Hrob 207
F-PiggyBac-4_Hrob 211
F-PiggyBac-5_Hrob 210
F-PiggyBac-6_Acal 207
F-PiggyBac-6_Bgla 207
Pokey
Pokey-PiggyBac-1_Dobt 209
Pokey-PiggyBac-1_Dpuli 209
Pokey-PiggyBac-2_Dobt 208
Pokey-PiggyBac-2_Dpuli 199

DDBD2

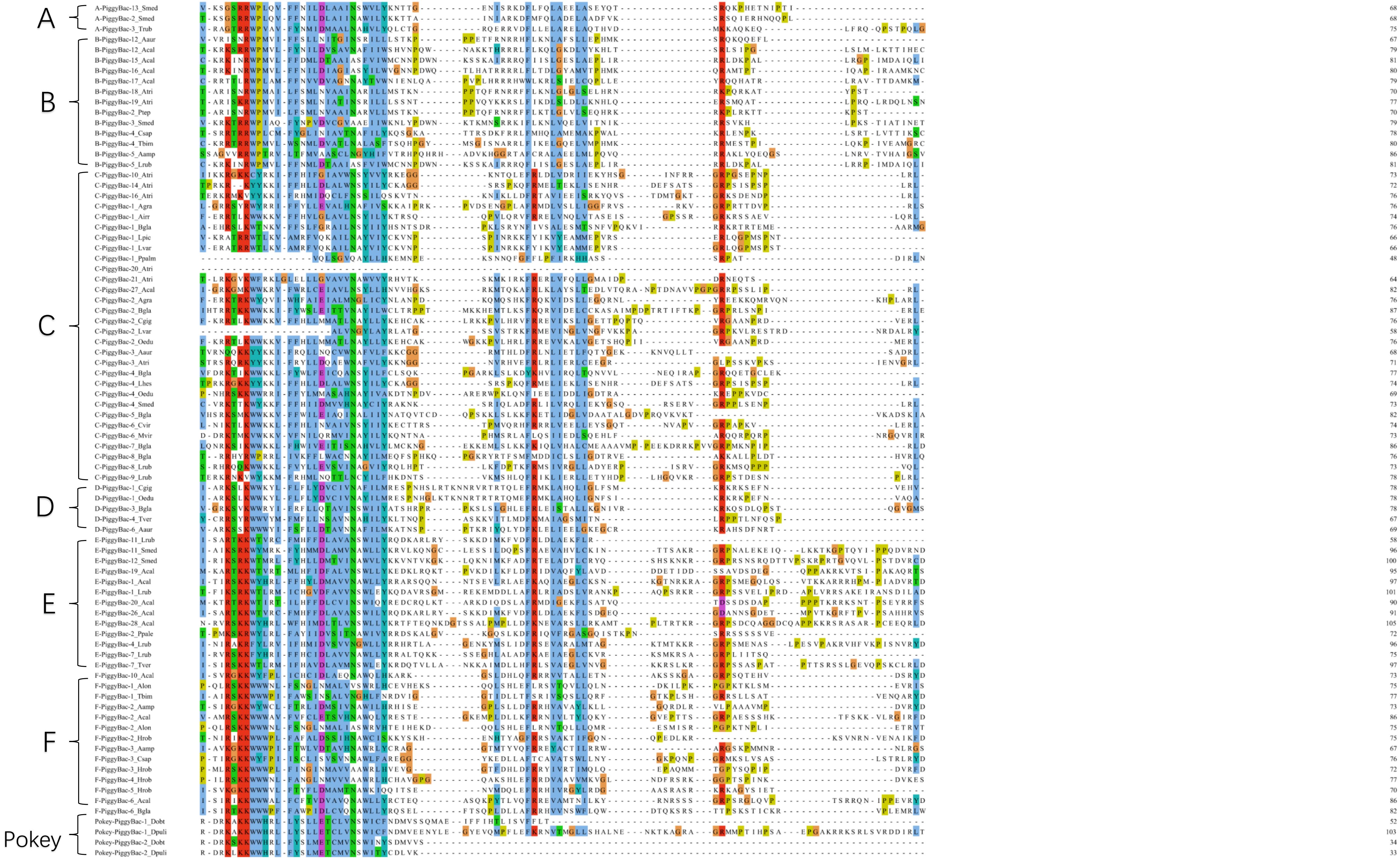
A
A-PiggyBac-13_Smed
A-PiggyBac-2_Smed
A-PiggyBac-3_Trub
B
B-PiggyBac-12_Aaur
B-PiggyBac-12_Acal
B-PiggyBac-15_Acal
B-PiggyBac-16_Acal
B-PiggyBac-17_Acal
B-PiggyBac-18_Atri
B-PiggyBac-19_Atri
B-PiggyBac-2_Ptep
B-PiggyBac-3_Smed
B-PiggyBac-4_Csap
B-PiggyBac-4_Tbim
B-PiggyBac-5_Aamp
B-PiggyBac-5_Lrub
C
C-PiggyBac-10_Atri
C-PiggyBac-14_Atri
C-PiggyBac-16_Atri
C-PiggyBac-1_Agra
C-PiggyBac-1_Airr
C-PiggyBac-1_Bgla
C-PiggyBac-1_Lpic
C-PiggyBac-1_Lvar
C-PiggyBac-1_Ppalm
C-PiggyBac-20_Atri
C-PiggyBac-21_Atri
C-PiggyBac-27_Acal
C-PiggyBac-2_Agra
C-PiggyBac-2_Bgla
C-PiggyBac-2_Cgig
C-PiggyBac-2_Lvar
C-PiggyBac-2_Oedu
C-PiggyBac-3_Aaur
C-PiggyBac-3_Atri
C-PiggyBac-4_Bgla
C-PiggyBac-4_Lhes
C-PiggyBac-4_Oedu
C-PiggyBac-4_Smed
C-PiggyBac-5_Bgla
C-PiggyBac-6_Cvir
C-PiggyBac-6_Mvir
C-PiggyBac-7_Bgla
C-PiggyBac-8_Bgla
C-PiggyBac-8_Lrub
C-PiggyBac-9_Lrub
D
D-PiggyBac-1_Cgig
D-PiggyBac-1_Oedu
D-PiggyBac-3_Bgla
D-PiggyBac-4_Tver
D-PiggyBac-6_Aaur
E
E-PiggyBac-11_Lrub
E-PiggyBac-11_Smed
E-PiggyBac-12_Smed
E-PiggyBac-19_Acal
E-PiggyBac-1_Acal
E-PiggyBac-1_Lrub
E-PiggyBac-20_Acal
E-PiggyBac-26_Acal
E-PiggyBac-28_Acal
E-PiggyBac-2_Ppale
E-PiggyBac-4_Lrub
E-PiggyBac-7_Lrub
E-PiggyBac-7_Tver
F
F-PiggyBac-10_Acal
F-PiggyBac-1_Alon
F-PiggyBac-1_Tbim
F-PiggyBac-2_Aamp
F-PiggyBac-2_Acal
F-PiggyBac-2_Alon
F-PiggyBac-2_Hrob
F-PiggyBac-3_Aamp
F-PiggyBac-3_Csap
F-PiggyBac-3_Hrob
F-PiggyBac-4_Hrob
F-PiggyBac-5_Hrob
F-PiggyBac-6_Acal
F-PiggyBac-6_Bgla
Pokey
Pokey-PiggyBac-1_Dobt
Pokey-PiggyBac-1_Dpuli
Pokey-PiggyBac-2_Dobt
Pokey-PiggyBac-2_Dpuli

CRD

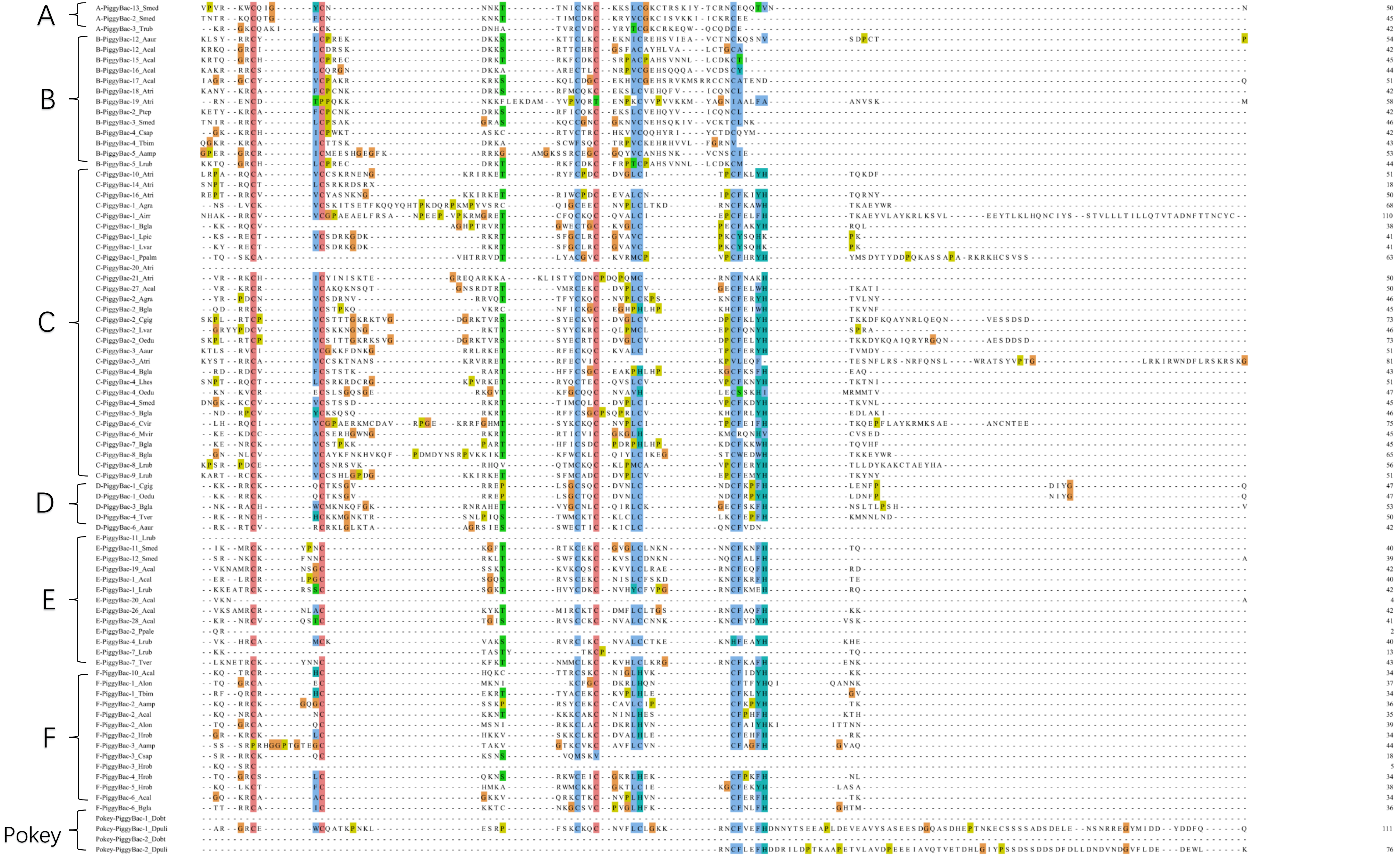
A
A-PiggyBac-13_Smed
A-PiggyBac-2_Smed
A-PiggyBac-3_Trub
B
B-PiggyBac-12_Aaur
B-PiggyBac-12_Acal
B-PiggyBac-15_Acal
B-PiggyBac-16_Acal
B-PiggyBac-17_Acal
B-PiggyBac-18_Atri
B-PiggyBac-19_Atri
B-PiggyBac-2_Ptep
B-PiggyBac-3_Smed
B-PiggyBac-4_Csap
B-PiggyBac-4_Tbim
B-PiggyBac-5_Aamp
B-PiggyBac-5_Lrub
C
C-PiggyBac-10_Atri
C-PiggyBac-14_Atri
C-PiggyBac-16_Atri
C-PiggyBac-1_Agra
C-PiggyBac-1_Airr
C-PiggyBac-1_Bgla
C-PiggyBac-1_Lpic
C-PiggyBac-1_Lvar
C-PiggyBac-1_Ppalm
C-PiggyBac-20_Atri
C-PiggyBac-21_Atri
C-PiggyBac-27_Acal
C-PiggyBac-2_Agra
C-PiggyBac-2_Bgla
C-PiggyBac-2_Cgig
C-PiggyBac-2_Lvar
C-PiggyBac-2_Oedu
C-PiggyBac-3_Aaur
C-PiggyBac-3_Atri
C-PiggyBac-4_Bgla
C-PiggyBac-4_Lhes
C-PiggyBac-4_Oedu
C-PiggyBac-4_Smed
C-PiggyBac-5_Bgla
C-PiggyBac-6_Cvir
C-PiggyBac-6_Mvir
C-PiggyBac-7_Bgla
C-PiggyBac-8_Bgla
C-PiggyBac-8_Lrub
C-PiggyBac-9_Lrub
D
D-PiggyBac-1_Cgig
D-PiggyBac-1_Oedu
D-PiggyBac-3_Bgla
D-PiggyBac-4_Tver
D-PiggyBac-6_Aaur
E
E-PiggyBac-11_Lrub
E-PiggyBac-11_Smed
E-PiggyBac-12_Smed
E-PiggyBac-19_Acal
E-PiggyBac-1_Acal
E-PiggyBac-1_Lrub
E-PiggyBac-20_Acal
E-PiggyBac-26_Acal
E-PiggyBac-28_Acal
E-PiggyBac-2_Ppale
E-PiggyBac-4_Lrub
E-PiggyBac-7_Lrub
E-PiggyBac-7_Tver
F
F-PiggyBac-10_Acal
F-PiggyBac-1_Alon
F-PiggyBac-1_Tbim
F-PiggyBac-2_Aamp
F-PiggyBac-2_Acal
F-PiggyBac-2_Alon
F-PiggyBac-2_Hrob
F-PiggyBac-3_Aamp
F-PiggyBac-3_Csap
F-PiggyBac-3_Hrob
F-PiggyBac-4_Hrob
F-PiggyBac-5_Hrob
F-PiggyBac-6_Acal
F-PiggyBac-6_Bgla
Pokey
Pokey-PiggyBac-1_Dobt
Pokey-PiggyBac-1_Dpuli
Pokey-PiggyBac-2_Dobt
Pokey-PiggyBac-2_Dpuli
