# Supplementary figures and images for: PiggyBac Transposon Mining in the Small Genomes of Animals
Source: Biology (Basel). 2023 Dec 31;13(1):24. doi: 10.3390/biology13010024 (PMC10813416; doi:10.3390/biology13010024)

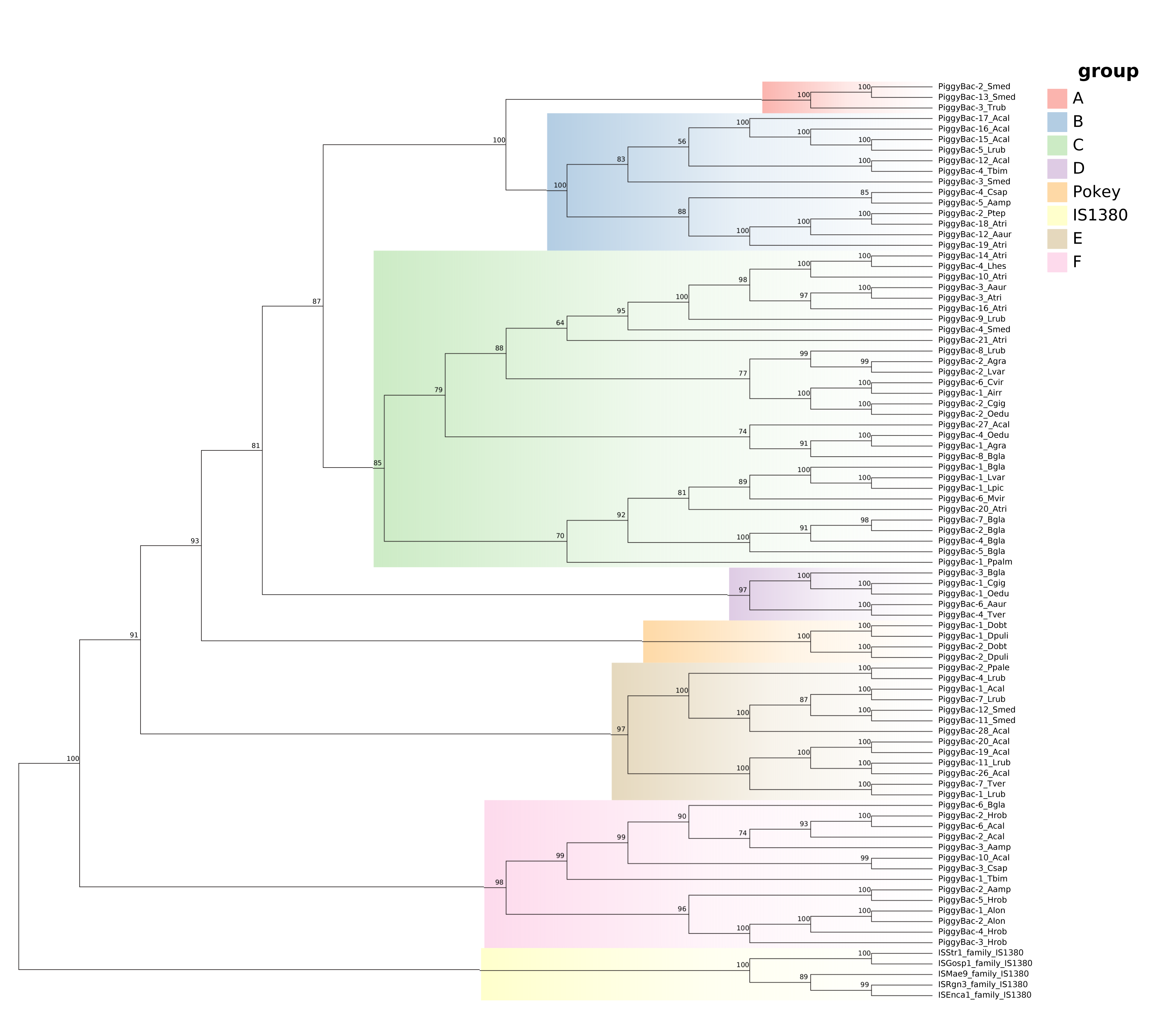

Supplement: Supplementary file 1 [file biology-13-00024-s001.zip › Supplement/Figure S2.tif]
